# Dry-hole analysis of exploratory wells in East Tiba-Natrun Area, NE Abu Gharadig Basin, Western Desert, Egypt: implications to petroleum exploration

**DOI:** 10.1038/s41598-026-64351-9

**Published:** 2026-08-24

**Authors:** Mona Ibrahim, Shawky Sakran, William Bosworth

**Affiliations:** 1 Department of Geology, Faculty of Science, Cairo University, Giza, 12613 Egypt; 2 PetroShahd Company, Zahraa El Maadi, Cairo, Egypt; 3 Jackson School of Geosciences, Department of Earth and Planetary Sciences, The University of Texas at Austin, University Station C9000, Austin, TX 78712-0254 USA

**Keywords:** East Tiba, Natrun, Western Desert, Dry-hole Analysis, Exploration Risk, Energy science and technology, Solid Earth sciences

## Abstract

Each non-productive or unsuccessful well is unique, tells its own story, and has distinctive reasons for being classified as a dry hole or lacking sufficient hydrocarbons. The objective of this study is to present a dry-hole analysis of three exploratory wells drilled in the East Tiba/Natrun study area, in the northeastern Western Desert of Egypt. Despite the Western Desert and the nearby Nile Delta provinces having proven hydrocarbon resources, these wells failed to deliver commercial discoveries. The analysis was conducted from a petroleum-system perspective, integrating geological, petrophysical, and well testing data to identify the causes of failure. The present study applies a systematic dry-hole evaluation workflow and develops a decision-tree framework for prospect selection and ranking. Each well was evaluated based on the effectiveness of the source rock, hydrocarbon migration pathways, reservoir quality, trap configuration, and seal integrity through comparative analysis. The key results and implications of this paper indicate that the primary causes of failure include seismic misinterpretation, trap failure, ineffective sealing mechanisms, and insufficient hydrocarbon charge due to lateral migration barriers or source maturity issues. In some cases, poor reservoir development or drilling through water-bearing zones in structurally complex settings contributed to the lack of success of the drilled wells. By identifying these failure mechanisms, the study offers valuable insights into exploration risk assessment and highlights the need for improved pre-drill modeling, seismic interpretation, and basin analysis. These conclusions highlight the significance of understanding dry-hole causes in mature hydrocarbon provinces like the Western Desert, as they reduce the risk of future dry holes and enhance exploration success rates in under-explored areas of the Western Desert, as well as the Nile Delta and Gulf of Suez in Egypt, and analogous sedimentary basins in North Africa and around the world.

## Introduction

**Fig. 1:**
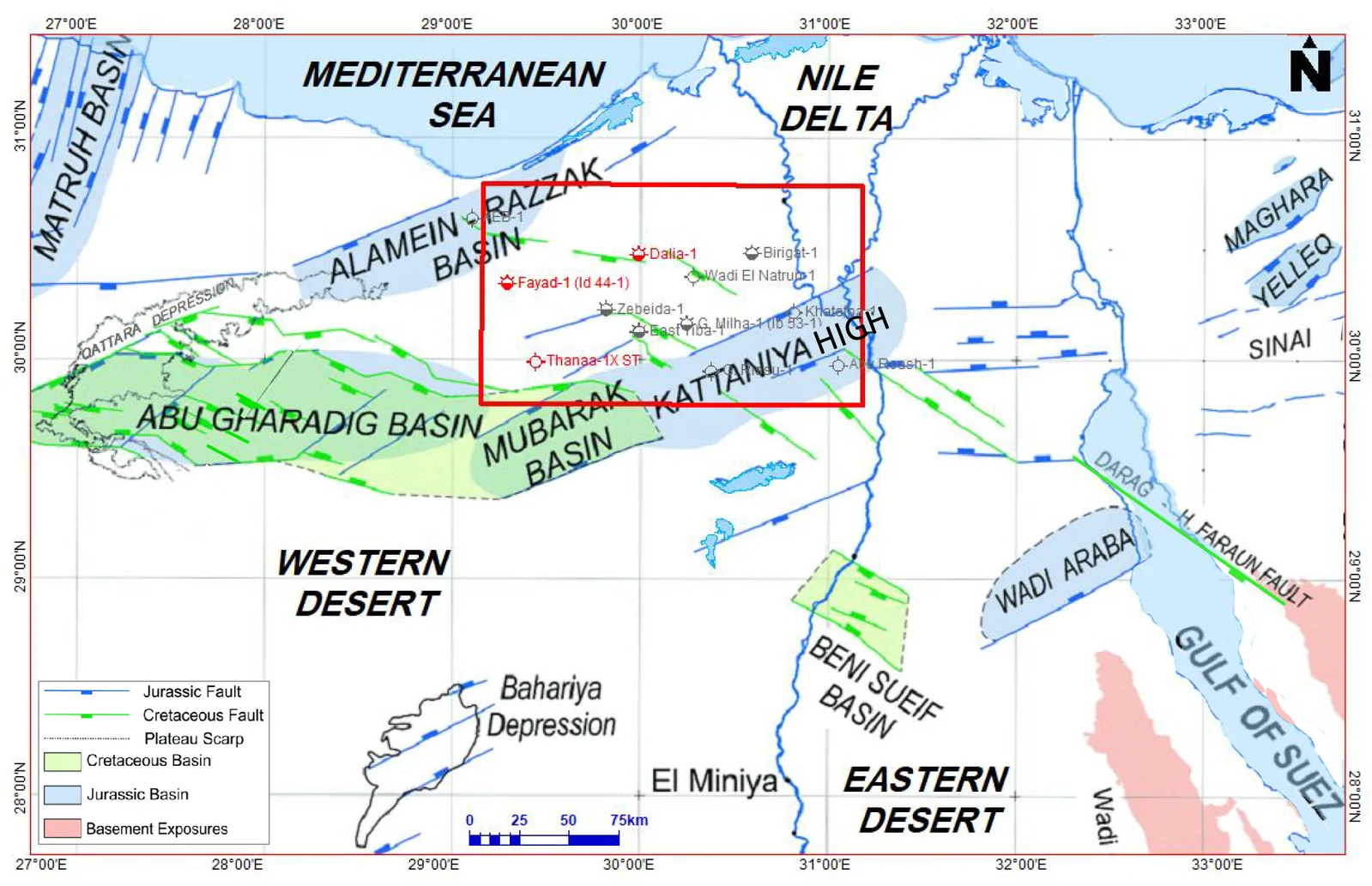
Regional Mesozoic sedimentary basins and faults of the northeastern part of the Western Desert, Egypt (after^2^). The location of the study area is indicated by a red rectangle. Wells used in this study are shown in red, while wells from legacy are shown in black, and more details are given in Fig. 2. Main Jurassic faults are shown as heavy blue lines, and main Cretaceous faults are shown as heavy green lines. Jurassic basins depocenters are shaded in light blue, and Cretaceous basins depocenters are shaded in light green. The Kattaniya High was a Jurassic depocenter then inverted later in the Late Cretaceous time. The Qattara depression, which is a surficial depression mostly with sabkha, is shown to the west of the study area.

**Fig. 2:**
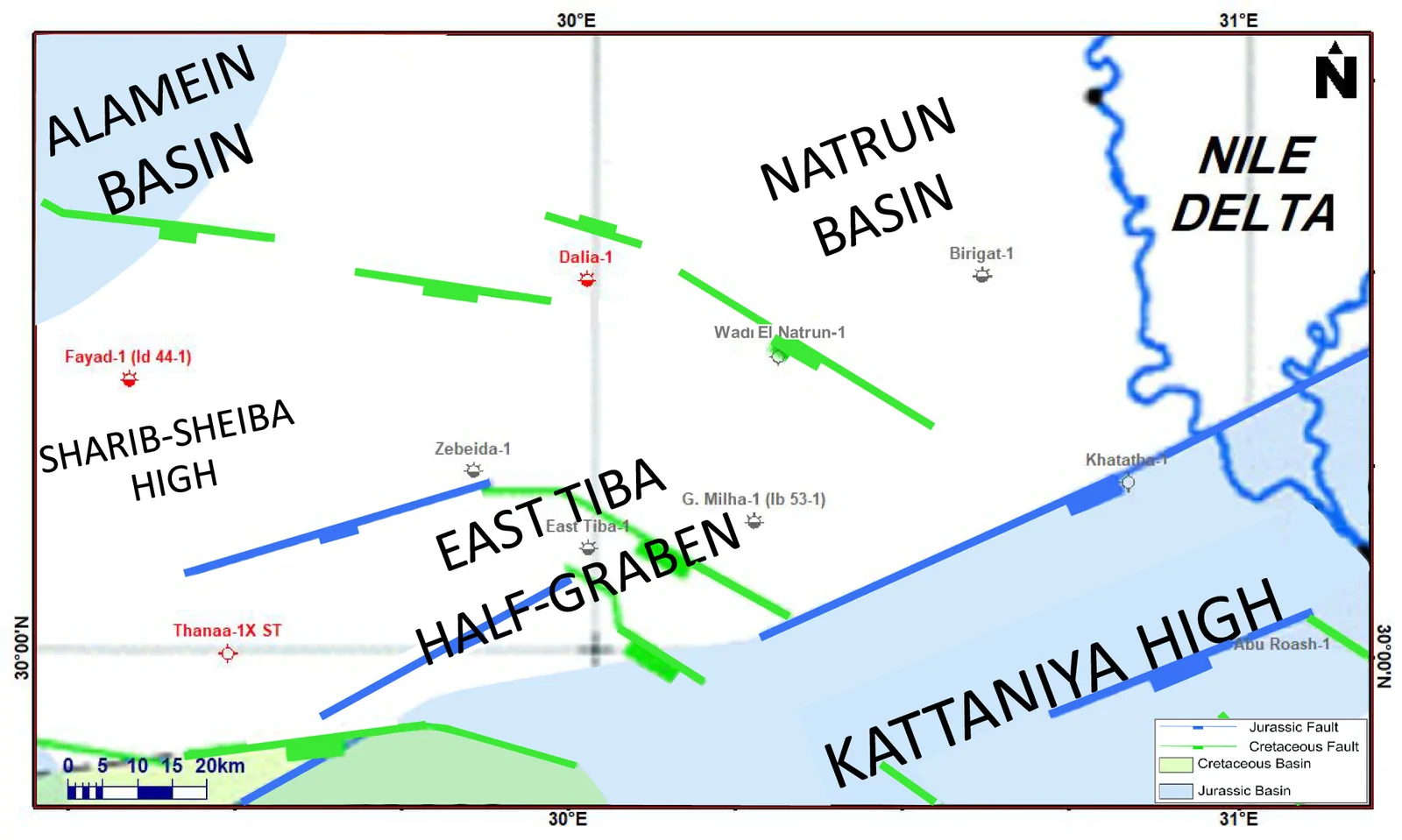
Details of the study area, comprising the Natrun basin and East Tiba half-graben. Wells used in the study are shown in red; other nearby important wells are shown in black.

The geological evolution of Egypt is characterized by multiple phases of basin formation, resulting in productive petroleum systems spanning the Paleozoic through Pliocene strata. The Western Desert is one of the important hydrocarbon mixed oil and gas provinces in North Africa, with most production concentrated in fault-related structural traps^1^. A complex subsurface geological history, diversity of structural settings, and a well-developed petroleum system characterize the northern part of the Western Desert. The study area lies within a tectonically complex region, onshore west of the Nile Delta between lat. 29° 48′ and 30° 48′ N and long. 29° 12′ and 31° 12′ E, covering about 22,375 km^2^ (Figs. 1 and 2). It is influenced by multiple rifting phases, resulting in varied basin architectures and trap styles. Over the past decades, numerous oil and gas discoveries have been made in this region, mainly from Cretaceous and Jurassic reservoirs.

Despite its proven hydrocarbon potential, exploration in the Western Desert continues to be challenged by geological uncertainties and structural complexities arising from limited geological data, variability in the quality and distribution of reservoir facies, the use of legacy 2D seismic data of poor imaging quality, especially for the deeper targets, and uncertainty in trap closure and seal integrity. These shortcomings have led to a significant number of dry holes. Low seismic quality remains one of the biggest obstacles to successful deep exploration, plus the historical focus on the Jurassic and Cretaceous targets based on previous exploration success^1^.

Understanding the reasons behind failures of some exploratory wells that failed to encounter commercial hydrocarbons despite favorable pre-drilling expectations is commonly referred to as “dry-hole analysis”. This analysis is critical for refining geological models, improving exploration risk assessment, and guiding future exploration. Unlike previous studies in the Western Desert of Egypt, which have mainly focused on specific reservoirs, localized geological interpretations, structural trends, reservoir characterization, petroleum-system analysis, or the evaluation of individual dry holes, the present study addresses this shortcoming by carrying out a comparative cross-well analysis of three exploratory dry holes (Fayad-1, Dalia-1, and Thanaa-1X). Each well was selected based on its strategic location and target reservoir objectives. The proposed methodology encompasses an integrated comparative and risk-based evaluation framework using seismic interpretation, structural analysis, petrophysical evaluation, drilling results, and petroleum–system assessment to evaluate the factors controlling dry-hole occurrence through a semi-quantitative exploration risk assessment. Although these three wells were dry or non-commercial, they provide valuable geological and operational data that enhance the understanding of failure mechanisms in the region and support the development of a decision-tree framework for exploration risk assessment. Furthermore, the study highlights the implications of using legacy 2D seismic data compared with updated 3D seismic data, providing practical guidance for improving seismic interpretation in structurally complex areas of the Western Desert.

The primary objective of this study is to identify and classify the causes of failure in each well, identify common exploration risks, and establish a practical framework for prospect evaluation. To achieve this objective, the workflow evaluates the principal exploration risk elements, including trap integrity, reservoir quality, seal effectiveness, source-rock charge, migration pathways, and operational performance.

## Geological setting

The Western Desert of Egypt, covering approximately 700,000 km^2^, constitutes a geologically diverse region that has undergone multiple tectonic events, shaping its current structural framework and stratigraphic configuration. The study area comprises the East Tiba and Natrun sub-basins (Fig. 2). It is bounded by several key structural features: the Sharib-Sheiba High to the west; the Kattaniya High (an inverted basin) to the south; and the Alamein Basin to the northwest (Fig. 2).

### Tectonic framework

Egypt, particularly the northern part of the Western Desert, has undergone several major tectonic phases and complex tectono-stratigraphic evolution spanning from the Precambrian to the Recent. Many authors have studied the tectonic evolution of the Western Desert of Egypt during the Mesozoic-Cenozoic (e.g.^2–10^).

The East Tiba-Natrun area represents a major depocenter at the eastern margin of the northern Western Desert, characterized by considerable Jurassic subsidence. The Natrun Basin is considered the thickest Jurassic basin in the Western Desert, with over 2700 m (~ 9000 ft) of Middle Jurassic sediments revealed from drilling, making the area unique in the Western Desert with a very high sedimentation rate, due to prolonged subsidence from Middle Bajocian to Bathonian times^11^.

An E-W trending positive feature near the Birigat-1 well (Fig. 2) separates the northern part of the basin with over 1500 m (~ 5000 ft) of Jurassic strata from the southern part with over 2700 m (~ 9000 ft) of shallow marine to deltaic Jurassic sediments. In the Lower Cretaceous, sediment thickness reached over 1800 m (~ 6000 ft) in the northern depocenter and about 1200 m (~ 4000 ft) in the southern sub-basin. This high continued to separate the northern and southern parts of the Natrun Basin during the Late Cretaceous and Cenozoic times^12^. Toward the northeast of the study area, shallow stratigraphic sections thin and transition into successions typical of the Nile Delta province, as proven by wells drilled near the Nile Delta.

The Mesozoic and Cenozoic of Egypt witnessed four main phases of deformation related to movements between the African and surrounding lithospheric plates: (1) rifting along the Neotethyan margin starting in the Permo-Triassic. In the Western Desert, this was most significant during the Middle to Late Jurassic and earliest Cretaceous, forming numerous extensional basins with predominantly E–W to ENE–WSW striking fault systems; (2) a shift to NE–SW extension in the mid-Early Cretaceous that overprinted the older structures with NW–SE striking faults. This change in extension direction was related to distant events along the evolving Atlantic rifts; (3) widespread NNW–SSE directed inversion in the Late Cretaceous starting at about 84 Ma (the “Santonian event”) that continued episodically and locally until the Late Eocene. This was due to a switch from opening of the Neotethys to convergence between Eurasia and Africa; and (4) Neogene opening of the Gulf of Suez-Red Sea rift system that continues to the present-day. Far-field effects of this youngest rifting are observed across the Western Desert both as NNW-SSE striking faults and intruded basaltic dikes^9–11,13^.

### Stratigraphic framework

**Fig. 3:**
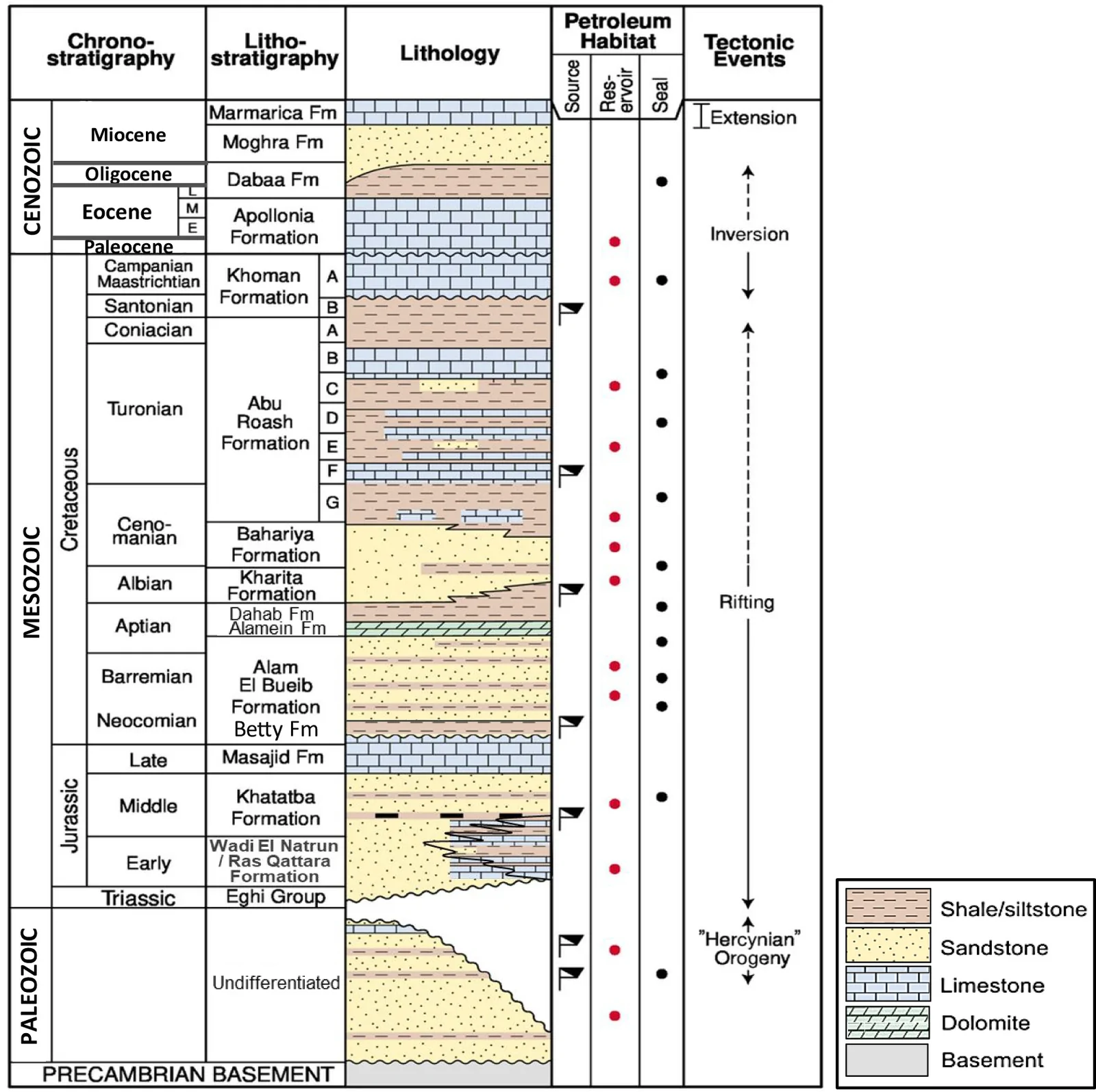
Litho-stratigraphic column, petroleum–system elements, and tectonic events of the northeastern part of the Western Desert (modified after^16,17^, and^12^).

The stratigraphic succession of the East Tiba-Natrun area records a complex stratigraphic column ranging from Precambrian basement to recent deposits (Fig. 3), providing significant exploration potential. The lithological sequences reflect multiple tectonic and sedimentary phases of alternating clastic and carbonate sequences associated with regional rifting, transgressions, and regressions due to global sea-level changes and variations in terrigenous input, which shaped the petroleum systems of the area. Many authors have described the stratigraphy of the study region in detail (e.g.^12,14–21^).

Precambrian basement rocks are the oldest penetrated unit in the region and are composed of igneous and metamorphic rocks. The Paleozoic succession is characterized by sandstone lithology, and it is undifferentiated and non-productive in the study area. Moreover, the Precambrian and Paleozoic rocks are not penetrated in many wells in the study area. The Mesozoic succession is characterized by the presence of Jurassic and Cretaceous sediments, which are considered the most important strata for hydrocarbon potential, formed through multiple large sedimentary cycles. These include transgressive and regressive phases, ranging from continental clastics to marine carbonates^22^. The Cenozoic sequences play a role as seals and overburden units that cap the underlying productive reservoirs. Variations in depositional facies, reservoir distribution, and seal effectiveness strongly influence petroleum–system performance and future prospectivity.

### Petroleum–system elements

**Fig. 4:**
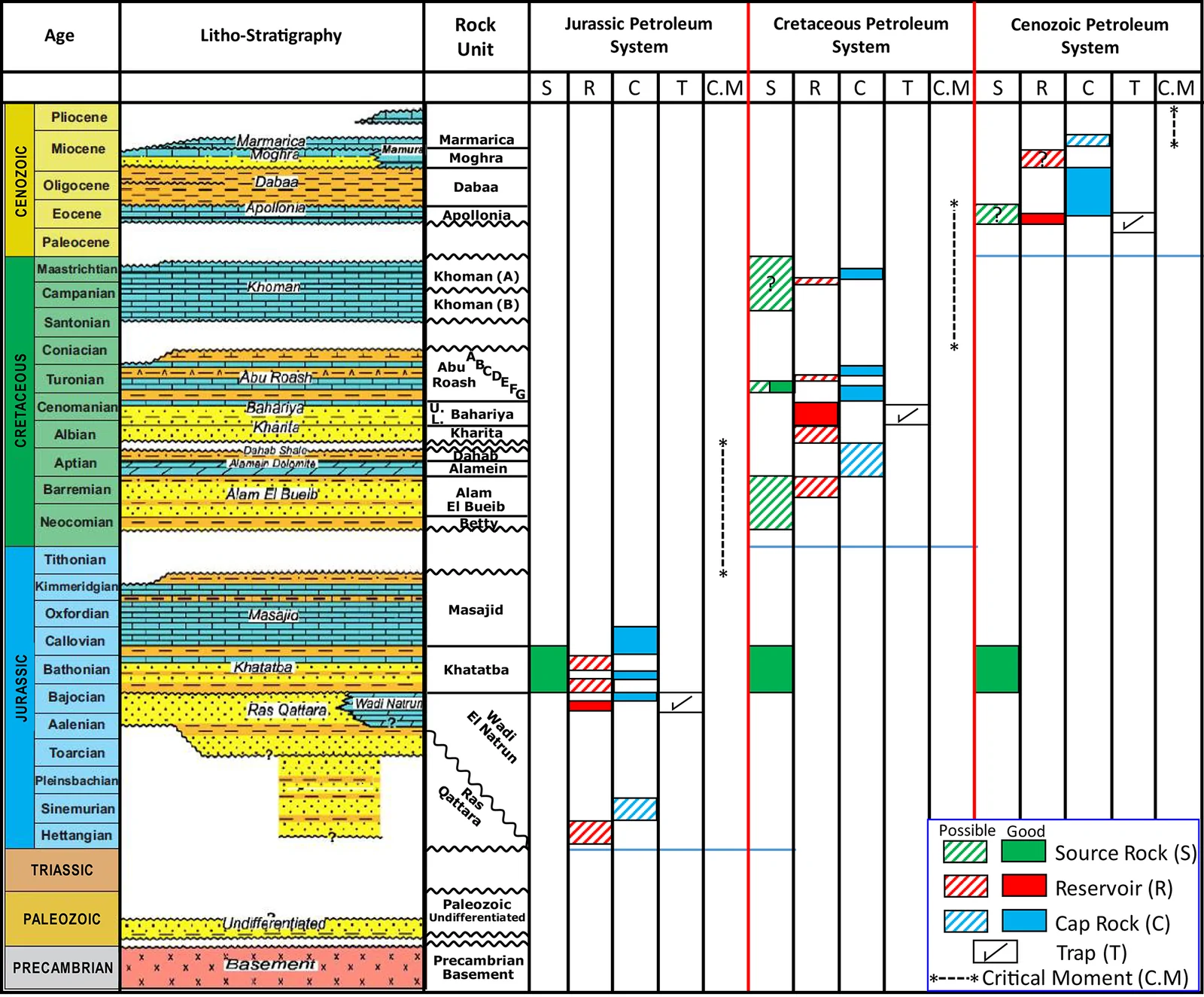
The litho-stratigraphic column of the East Tiba-Natrun area, Western Desert, and interpreted Jurassic, Cretaceous, and Cenozoic petroleum systems (after^25,26^).

The main elements of a petroleum system are the source, reservoir, seal, and overburden rocks. Two key processes manage this system: trap formation and hydrocarbon generation, migration, and accumulation. These elements and processes must be correctly placed in time and space, allowing the organic matter in the source rock to be converted into a petroleum accumulation. According to^23,24^, a petroleum system is considered to exist wherever these essential elements and processes are confirmed or have a reasonable likelihood of occurring. A detailed understanding of these elements is essential for reducing exploration risk and preventing dry holes. The Western Desert is one of the principal petroleum provinces of North Africa, hosting multiple petroleum systems developed from Paleozoic to Cenozoic time. The Western Desert includes three main petroleum systems: Jurassic, Cretaceous, and Cenozoic, each with distinct associations of reservoirs, source rocks, traps, and seals (Fig. 4)^25,26^.

The Jurassic petroleum system is characterized by the Khatatba Formation, the shale units of which represent a major source rock in the region, feeding the formed traps with migrated hydrocarbons^27,28^. It includes the sandstone-rich Upper and Lower Safa Members, which act as reservoir rocks. The overlying Masajid Formation (carbonates) provides excellent top seal. The underlying Ras Qattara Formation and sandstone intervals within the Wadi El Natrun Formation (carbonates) also act as reservoir rocks (Fig. 4). Sourcing these deeper units from the Khatatba requires appropriate structural juxtaposition^29^.

The Cretaceous petroleum system includes the Alam El Bueib Formation (sandstones and siltstones) acting as a key reservoir unit, although its effectiveness is sometimes limited by lateral sealing problems. It is also considered a potential source rock due to the presence of shale-rich intervals^30,31^. The Alamein Formation, which is predominantly dolostone also serves as a target reservoir in certain parts of the study area, while the Dahab Formation with shaly content, acts as a top seal (Fig. 4). An additional component of the Cretaceous petroleum system involves the Kharita and Bahariya formations, which act as major reservoirs, particularly within the East Tiba half-graben. The overlying Abu Roash “G” Member is composed mainly of shale and carbonate in the East Tiba area, where it acts as an effective top and lateral seal for the Bahariya reservoir. Northward, lateral facies change results in increased dolomitization and the development of sandstone bodies, causing the Abu Roash “G” Member to transition from a regional seal into a potential reservoir unit in the Natrun Basin and adjacent areas. Furthermore, to the southwest of the study area, the Abu Roash “G” Member forms a productive Turonian reservoir comprising two hydrocarbon-bearing zones with good reservoir properties, demonstrating its significant reservoir potential where suitable facies are developed^32^. Other members of the Abu Roash Formation contribute to this system as possible reservoirs, particularly Abu Roash “E” sandstones. The Abu Roash “F” Member is acting as a mature source rock in the depocenters and immature to semi-mature on their margins^12,33^. The Abu Roash “D” and “C” Members generally play the role of top seal, although the Abu Roash “C” contains reservoir-quality sandstone facies in some adjacent areas. The Abu Roash “B” Member forms a carbonate reservoir in some parts of the Western Desert, but not within the Natrun Basin or East Tiba half-graben. The Abu Roash “A” Member is composed of siliciclastics with carbonate streaks, and acts as a cap rock, while the overlying Khoman Formation functions as a regional seal and is also considered a possible source rock^34^ (Fig. 4).

The Cenozoic petroleum system includes the Apollonia Formation, which serves as both an in-situ source rock when organic matter reaches sufficient thermal maturity at suitable burial depths, and it is an unconventional reservoir in some areas^25^. This Formation has source-rock characteristics in the lithologies of argillaceous limestone, with good, mainly oil-prone source-rock quality. However, this unit is thermally immature throughout most of the northern Western Desert, especially at the basin margins. At the same time, in the depocentres that have not yet been penetrated, it could be mature enough for hydrocarbon generation^33^. The Dabaa Formation, rich in shale, provides a regional cap rock for this system (Fig. 4).

The Western Desert petroleum province includes hydrocarbon traps of a diversity of structural styles; their geometries vary from basin to basin^35^. The trap styles for the Jurassic, Cretaceous, and Cenozoic petroleum systems are generally structural (folding or faulting), particularly tilted fault blocks as 3-way closures, and anticlines as 4-way closures. However, stratigraphic pinch-outs and combination traps also occur. The main mechanisms of hydrocarbon migration are lateral along carrier beds and vertical along fault planes from mature kitchen areas into structural highs. Some faults also seal and trap hydrocarbons, indicating that their role in the hydrocarbon systems is complex and may vary through time.

## Database and methodology

**Fig. 5:**
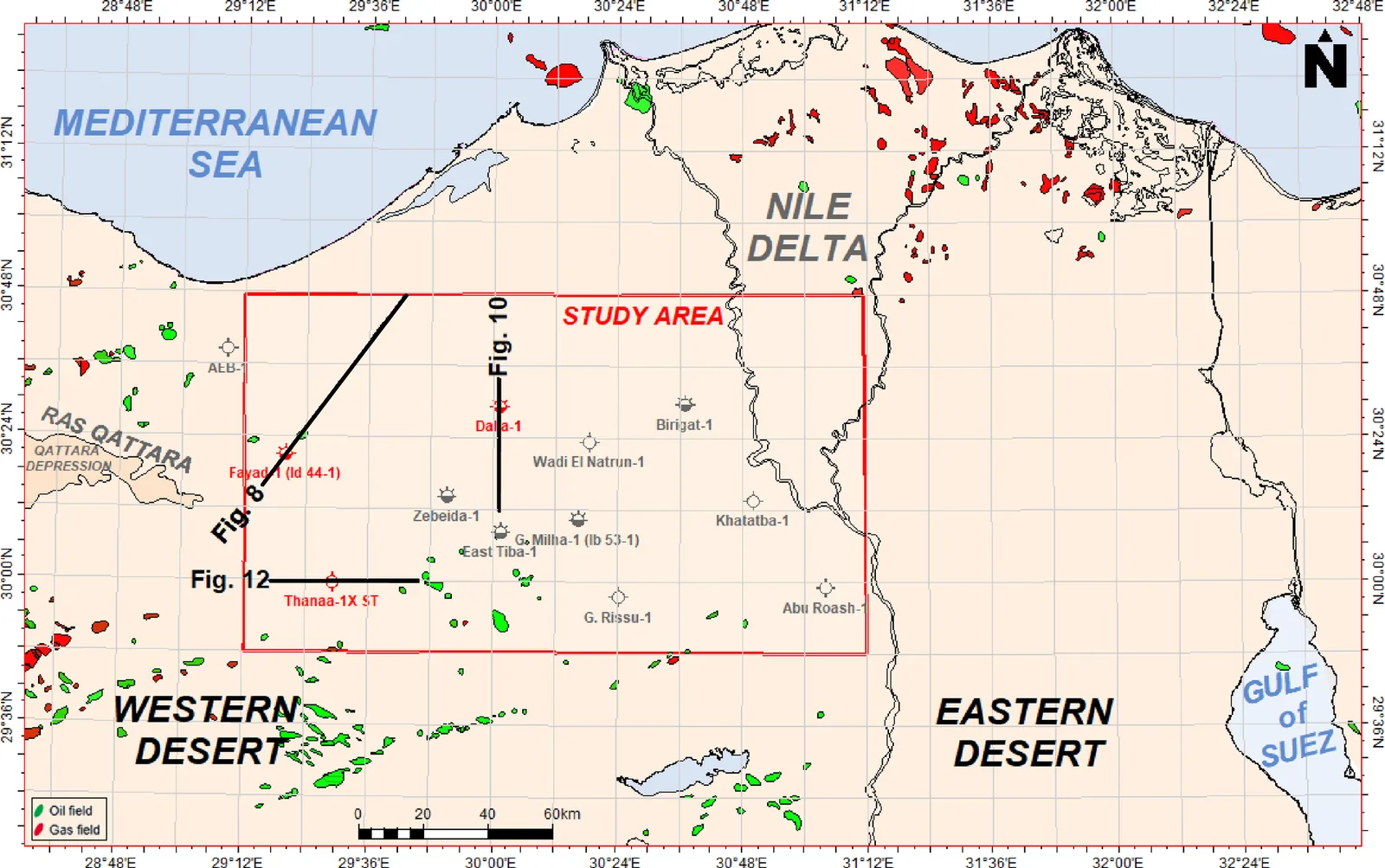
Location map of the studied wells with display of the seismic lines in black, and nearby producing fields (green color for oil and red color for gas producing wells). See Fig. 1 for more regional settings.

This study employs an integrated dry-hole analysis approach, utilizing geological, geophysical, petrophysical, and operational data from three exploratory wells drilled in the East Tiba-Natrun area. Information from nearby producing fields was incorporated whenever possible (Fig. 5). The goal is to evaluate the causes of well failure and to extract exploration insights relevant to the region’s petroleum–system framework.

### Database

Published regional studies were used to identify the depositional history and unconformities (hiatuses and erosion events) within the local stratigraphic section. Stratigraphic correlation panels were constructed and then integrated with the structural framework and tectonic maps. The available well data (Table 1) include formation tops, lithological descriptions from ditch cuttings, mud logs, composite logs, electric logs (gamma ray, resistivity, density, neutron), petrophysical evaluation results, drill stem test (DST) results, and drilling reports. The seismic dataset comprised a combination of 2D vintage seismic lines and 2D sections extracted from 3D seismic volumes, with time-depth (TD) listings. These data were subsequently interpreted to evaluate the structural configuration, stratigraphic framework, and distribution of reservoir, seal, and source rock intervals within the studied wells in the study area. The geochemical and petrographic dataset included regional source rock maturity profiles, organic richness estimates, and geochemical logs derived from cuttings samples.

**Table 1:** Available data of the studied wells.

Well name	Seismic line	Formation tops	VSP–TD listing	Electric logs					Petrophysical evaluation	Geochemical evaluation
				GR	Resistivity	Neutron	Density	Sonic DT		
Fayad-1	√	√		√	√	√	√	√		√
Dalia-1	√	√	√	√	√	√	√	√	√	√
Thanaa-1X ST	√	√	√	√	√		√	√	√	√

### Methodological approach

**Fig. 6:**
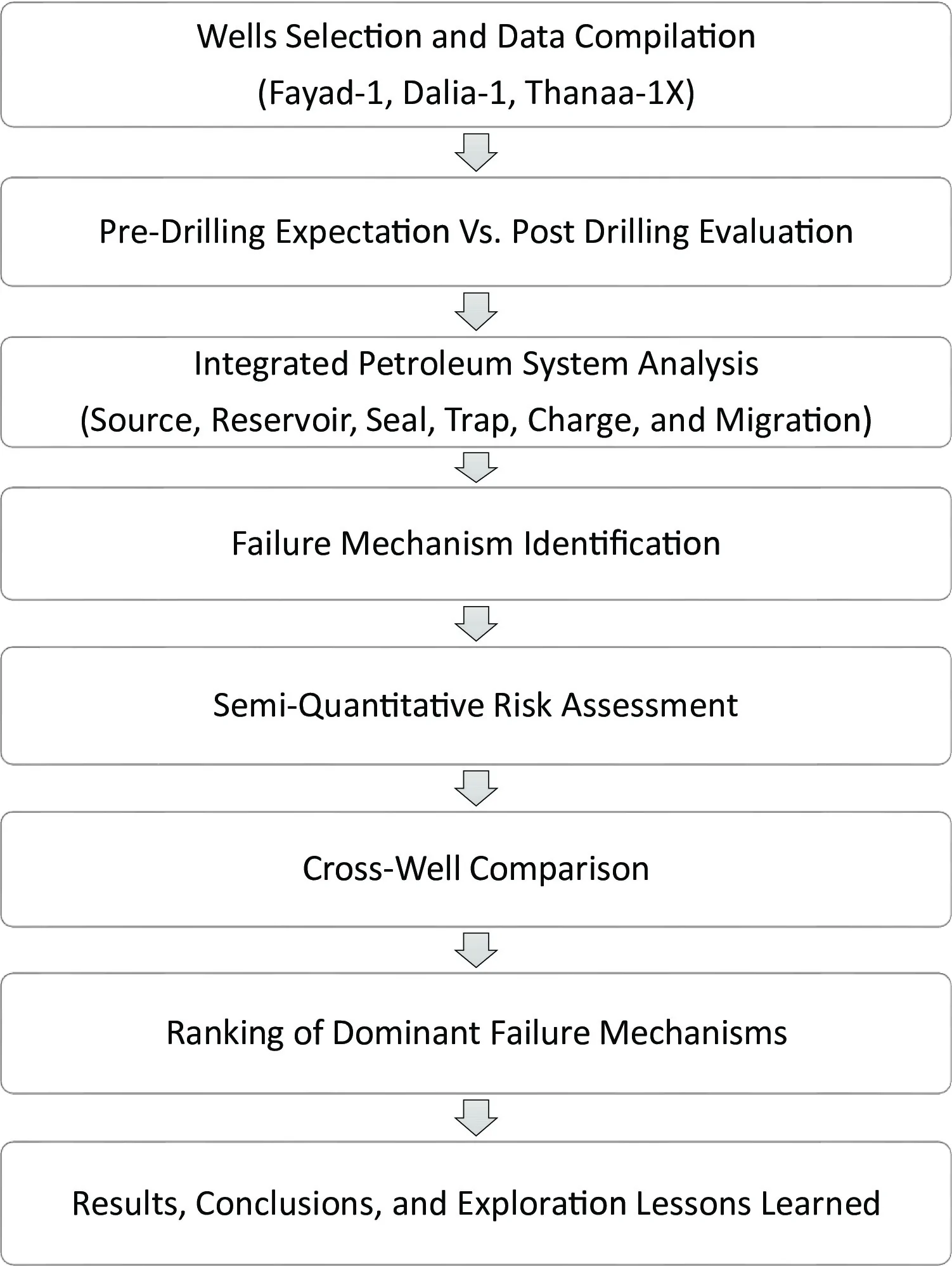
Methodological workflow adopted for the comparative analysis of three dry holes in the Western Desert of Egypt. The workflow integrates pre-drilling and post-drilling evaluation, petroleum–system analysis, failure mechanism identification, semi-quantitative risk assessment, and cross-well comparison to determine the dominant causes of failure and develop exploration lessons for future prospect evaluation and risk reduction.

**Fig. 7:**
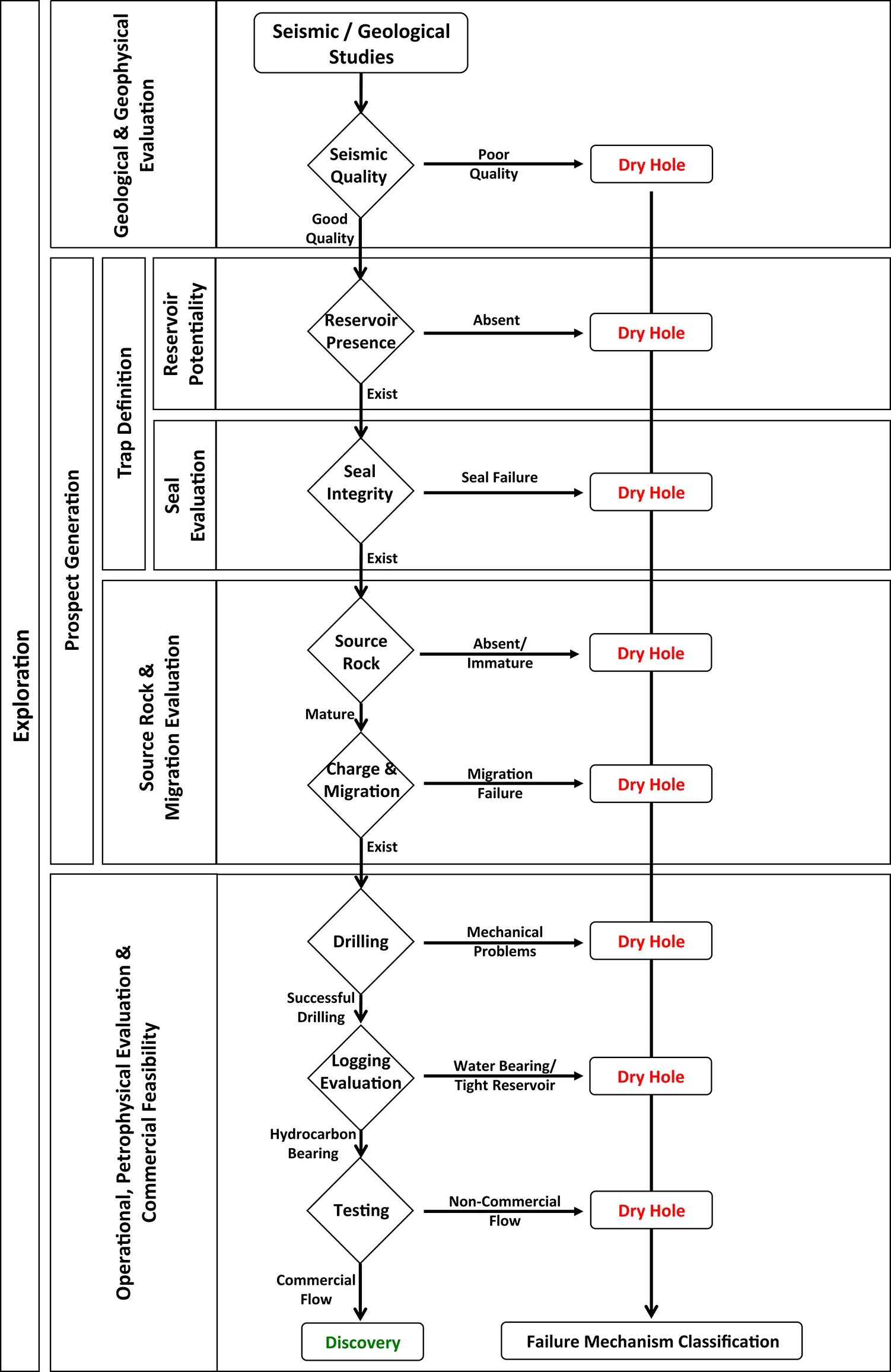
Conceptual decision-tree framework for dry-hole analysis and failure mechanism classification. The workflow evaluates geological, geophysical, petroleum system, operational, and commercial factors that control exploration success. The workflow identifies potential causes of dry-hole results, including seismic uncertainty, reservoir absence, seal failure, source-rock immaturity, charge/migration failure, drilling problems, unfavorable reservoir quality, and non-commercial testing results. The framework was applied to classify failure causes in the studied wells and to support cross-well comparison and exploration risk assessment.

The systematic workflow (Fig. 6) ensures that both geological and technical causes of failure are considered, providing a reliable understanding of subsurface risks and opportunities in the northeastern Western Desert. The applied dry-hole analysis framework (Fig. 7) addresses the failure cause, whether related to structure, stratigraphy and reservoir, seal efficiency, source rock charge/migration pathways, and operational /mechanical factors. Each well was evaluated in terms of the essential elements and processes of a petroleum system, as well as the uncertainties associated with drilling and data interpretation^23,24^.

The methodological contribution of this study is the application of a unified dry-hole assessment workflow to three representative exploratory wells in a common geological setting in the Western Desert of Egypt. Each well was evaluated using the same sequence of analyses, including structural interpretation, reservoir assessment, seal evaluation, source-rock charge analysis, and drilling results. This workflow enables direct cross-well comparison, identification of common failure mechanisms, and ranking of exploration risks, providing a framework for dry-hole investigations. The results are then incorporated into a practical decision-tree workflow (Fig. 7) that can be applied during prospect maturation and exploration risk assessment. The methodological approach included the following steps:

Step 1: Well selection and data compilation.

The selection of three key exploratory wells (Fayad-1, Dalia-1, and Thanaa-1X) was based on their geographic distribution, target stratigraphic reservoirs, the causes of their dry-hole status, and the availability of subsurface data. The selected wells tested the structural play concepts commonly employed in the Natrun–East Tiba area. Geological, geophysical, petrophysical, drilling, and test data were compiled to establish a consistent database for the subsequent analyses.

Step 2: Comparison between pre-drilling expectations versus post-drilling results.

The pre-drilling objectives, expected reservoir zones, and the interpreted trap geometries were compared with the actual drilling results. Post-drilling data, including seismic reinterpretation calibrated with well data, drilling reports, mud logs, and available petrophysical data, were used to evaluate deviation from the pre-drilling expectations and to identify the main causes of failure.

Figure 7 summarizes the proposed conceptual decision-tree workflow used to classify dry-hole mechanisms. The workflow begins with the evaluation of geological and geophysical data, including seismic data quality, structural interpretation, and prospect definition. Structural configuration and the stratigraphic framework are first evaluated from seismic data, where poor seismic quality may lead to interpretation uncertainty and early exploration failure. Where seismic confidence is adequate, prospect generation proceeds through evaluation of trap geometry, reservoir distribution, seal integrity, source-rock maturity, hydrocarbon generation, and migration pathways. Reservoir presence and quality are evaluated using lithological and petrophysical data, with emphasis on potential sandstones and fractured carbonate reservoirs, as inadequate reservoir properties may prevent hydrocarbon accumulation even within structurally valid traps. Seal integrity, which represents a critical control on hydrocarbon preservation, is subsequently evaluated because fault reactivation, facies changes, or ineffective top and lateral seals may result in hydrocarbon leakage and trap failure and, as a consequence, a dry hole. Source rock evaluation includes source-rock presence, organic matter richness “total organic carbon (TOC) content”, thermal maturity, hydrocarbon-generation potential, and migration pathways to the evaluated traps. The absence of mature source rocks or ineffective migration pathways may also result in exploration failure. Fault-seal analysis is integrated with structural interpretation and well data to evaluate trap integrity and hydrocarbon preservation, following methodologies commonly applied in the northern Western Desert^36^. Finally, operational and commercial factors, including drilling performance, well logging, petrophysical evaluation (net reservoir, net pay, porosity, and permeability where available), well testing, and reservoir producibility, are evaluated to determine whether identified hydrocarbons are technically and commercially recoverable. Failure of any critical petroleum-system element may result in a dry hole, whereas successful evaluation of all elements leads to a commercial hydrocarbon discovery.

Step 3: Integrated petroleum–system analysis (elements and processes).

Each well was evaluated with respect to the key petroleum–system elements and processes, including source rock, reservoir, seal, trap, hydrocarbon charge, and migration pathways. Trap and structural configuration were evaluated with respect to fault geometry, structural closure, seismic interpretation, and fault-seal analysis. Reservoir presence and quality were evaluated through sandstone development, available petrophysical properties (effective porosity, water saturation, volume of shale, net-reservoir thickness, and net pay where it is available), and the effects of diagenesis on reservoir performance. Seal efficiency was examined by analyzing the presence, continuity, and integrity of both top and lateral sealing units. Source rock evaluation focused on the vicinity of the well to mature source intervals and the timing of hydrocarbon generation relative to trap formation. Migration pathways were investigated by considering structural connectivity and potential migration barriers that could impede hydrocarbon charge.

The petrophysical cut-off values were selected on a formation-specific basis to account for variations in lithology and reservoir characteristics. The adopted cut-off criteria were derived from processed well-log interpretations, calibrated using nearby producing field analogues, and are consistent with published studies of analogous reservoirs in the Western Desert^36^. Minor differences in porosity cut-offs were applied to reflect local reservoir quality variations between sandstone and carbonate reservoirs. The pay cut-offs are summarized in Table 2.

**Table 2:** Petrophysical cut-off values used for reservoir evaluation.

Parameter	Cut-off value					
	Bahariya	Kharita	Alamein	AEB	Masajid	Khatatba
Effective porosity (PHIE) %	≥ 8–10*	≥ 8.1	≥ 5	≥ 9	≥ 5	≥ 8
Water saturation (Sw) %	≤ 50	≤ 50	≤ 60	≤ 50	≤ 50	≤ 50
Volume of shale (Vsh) %	≤ 35	≤ 50	≤ 50	≤ 50	≤ 50	≤ 45

*The reported cut-off values represent the range adopted during the original petrophysical interpretation of the studied wells. Minor variations between wells reflect formation-specific calibration and local reservoir characteristics.

Step 4: Failure mechanism identification.

Based on the integrated petroleum–system evaluation, each well was classified according to its failure mechanism into trap, reservoir, seal, and charge/migration failures, in addition to operational and non-economic failures. Operational failure refers to wells that were abandoned due to drilling or completion-related mechanical problems. Non-economic failure describes cases where hydrocarbons are present but do not meet commercial criteria. This includes accumulations with insufficient hydrocarbon volumes, low productivity, and/or unfavorable economic conditions, such as high development costs and low oil prices^23,37,38^.

Step 5: Semi-quantitative exploration risk assessment.

**Fig. 8:**
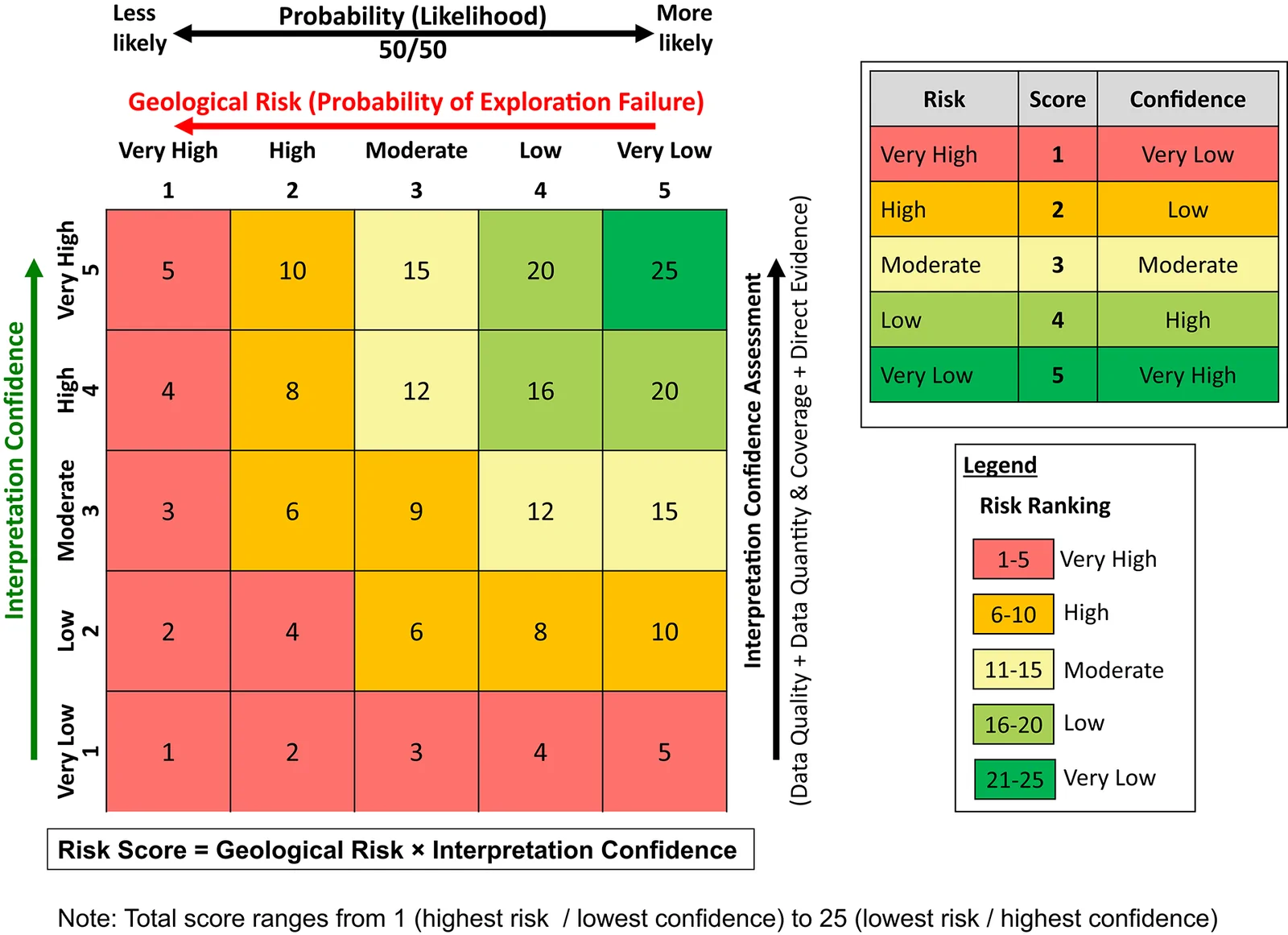
Semi-quantitative exploration risk-confidence matrix for dry-hole evaluation (modified after^38^). Geological risk was evaluated independently from interpretation confidence and integrated to calculate an exploration score ranging from 1 (highest geological risk/lowest interpretation confidence) to 25 (lowest geological risk/highest interpretation confidence). Interpretation confidence was derived from data quality, data quantity and coverage, and direct evidence. The matrix provides the framework used to compare exploration uncertainty between the studied wells.

To improve the objectivity of dry-hole classification, a semi-quantitative exploration risk assessment was developed to evaluate the principal petroleum-system elements and operational performance. The evaluated geological risk elements include structure/trap integrity, reservoir quality, seal effectiveness, source-rock (charge and migration), and operational performance. Each element was evaluated using the criteria summarized in Table 3, then assigned a geological risk score ranging from 1 (very high risk) to 5 (very low risk). The interpretation confidence evaluates the data independently from geological risk using three elements: data quality, data quantity and coverage, and direct evidence (Table 4), then assigns an interpretation confidence score by taking the average score of these three evaluated elements, the score ranging from 1 (very low confidence) to 5 (very high confidence). The total exploration score is calculated from the multiplication of both the geological risk score and the interpretation confidence score, on a scale from 1 to 25, where 1 is the highest geological risk associated with the lowest confidence, and 25 is the lowest geological risk associated with the highest interpretation confidence (Fig. 8).

**Table 3:** Semi-quantitative risk scoring criteria used for petroleum-system elements and operational performance.

Element	Score	Description
Structure/trap integrity	1	No structural closure or breached trap
	2	Poorly defined closure with high uncertainty
	3	Moderately defined closure with moderate structural uncertainty
	4	Well-defined closure
	5	Well-defined and fully closed trap with high confidence
Reservoir quality	1	Reservoir absent
	2	Tight reservoir with negligible net pay
	3	Marginal reservoir quality with limited net pay
	4	Good reservoir quality
	5	Excellent reservoir quality with effective porosity and net pay
Seal effectiveness	1	Seal failure
	2	Poor seal with leakage risk
	3	Partial sealing
	4	Effective seal
	5	High effective top and lateral seal with high confidence
Source rock (charge and migration)	1	No evidence of hydrocarbon charge
	2	Limited evidence of hydrocarbon charge or migration
	3	Uncertain hydrocarbon charge or migration
	4	Proven hydrocarbon charge
	5	Proven charge and effective migration pathways
Operational performance	1	Major drilling failure preventing evaluation
	2	Significant drilling problems
	3	Moderate operational issues
	4	Minor operational issues
	5	Successful drilling, well logging, and testing

Seismic resolution was not evaluated as an independent exploration element but was considered a confidence factor in structural interpretation and trap assessment.

**Table 4:** Semi-quantitative confidence scoring criteria.

Element	Score	Description
Data quality	1	Very Poor: legacy 2D seismic, uncalibrated well logs, or poor-quality vintage seismic data with limited interpretation
	2	Poor: low-resolution seismic or well-log data with significant imaging limitations and uncertainty
	3	Moderate: available data with moderate quality; some noise or imaging challenges are present
	4	Good: high-resolution 3D seismic integrated with calibrated well logs, providing reliable subsurface interpretation
	5	Excellent: high-density 3D seismic integrated with image logs, core data, and calibrated well logs, providing excellent interpretation confidence
Data quantity and coverage	1	Very Sparse: only regional 2D seismic data are available, with interpretation relying mainly on regional trends and distant data extrapolation
	2	Sparse: limited data coverage, such as distant 2D seismic lines and widely spaced exploration wells which cannot tie with each other, so provide poor structural control
	3	Moderate: adequate coverage through partial or legacy 3D seismic supported by a limited number of nearby offset wells
	4	Dense: full 3D seismic coverage with a reasonable density of exploration or appraisal wells
	5	Abundant: high-density 3D seismic covering the entire study area and integrated with many wells
Direct evidence	1	None/Theoretical: interpretation is based only on conceptual geological models or distant analogues without local supporting evidence
	2	Indirect/Weak: interpretation relies on weak indicators or possible geological interpretations
	3	Inferred: interpretation is supported by indirect evidence such as seismic attributes without direct calibration
	4	Direct: interpretation is supported by direct subsurface evidence, including well logs, core data, pressure data, or fluid inclusion analyses
	5	Proven: interpretation is validated by successful well tests, production data from the same reservoir, or Direct Hydrocarbon Indicators (DHI)

Step 6: Cross-well comparison.

The interpreted failure mechanisms and exploration risk scores were compared among the studied wells to identify common geological factors, repeated exploration risks, and variations in petroleum–system performance across the study area.

Step 7: Ranking of dominant failure mechanisms.

The identified failure mechanisms were ranked according to their relative occurrence and significance to determine the dominant causes of dry holes within the study area. This ranking provides a basis for prioritizing exploration risks in future drilling campaigns.

Step 8: Results, conclusions, and exploration lesson-learned.

The integrated geological, geophysical, petrophysical, and exploration risk analyses were integrated and compared to identify common exploration risks and the main causes of dry-hole failure, and to develop recommendations for reducing future drilling risk in the northeastern part of the Western Desert. In addition, structural elements were integrated with seismic interpretations to validate the geological models.

## Results

This section presents the integrated evaluation of the three dry exploratory wells using post-drilling data, seismic reinterpretation, petrophysical evaluation, and available geochemical data. The causes of failure were subsequently evaluated using the semi-quantitative exploration-risk framework described in the methodology.

### Fayad-1 (Id 44 − 1) well

The Fayad-1 well was drilled in 1975 in the western part of the study area on old vintage seismic data to test the hydrocarbon potential of Jurassic and Cretaceous reservoirs. Pre-drilling expectations assumed effective hydrocarbon charge from the regionally mature Jurassic Khatatba Formation, the principal source rock across much of the Western Desert. The well reached a total depth (TD) of 3600 m (~ 11,811 ft) within the Cretaceous Alam El Bueib (AEB) Formation; however, no hydrocarbon shows were reported during drilling, and the well was initially classified as a dry hole.

**Fig. 9:**
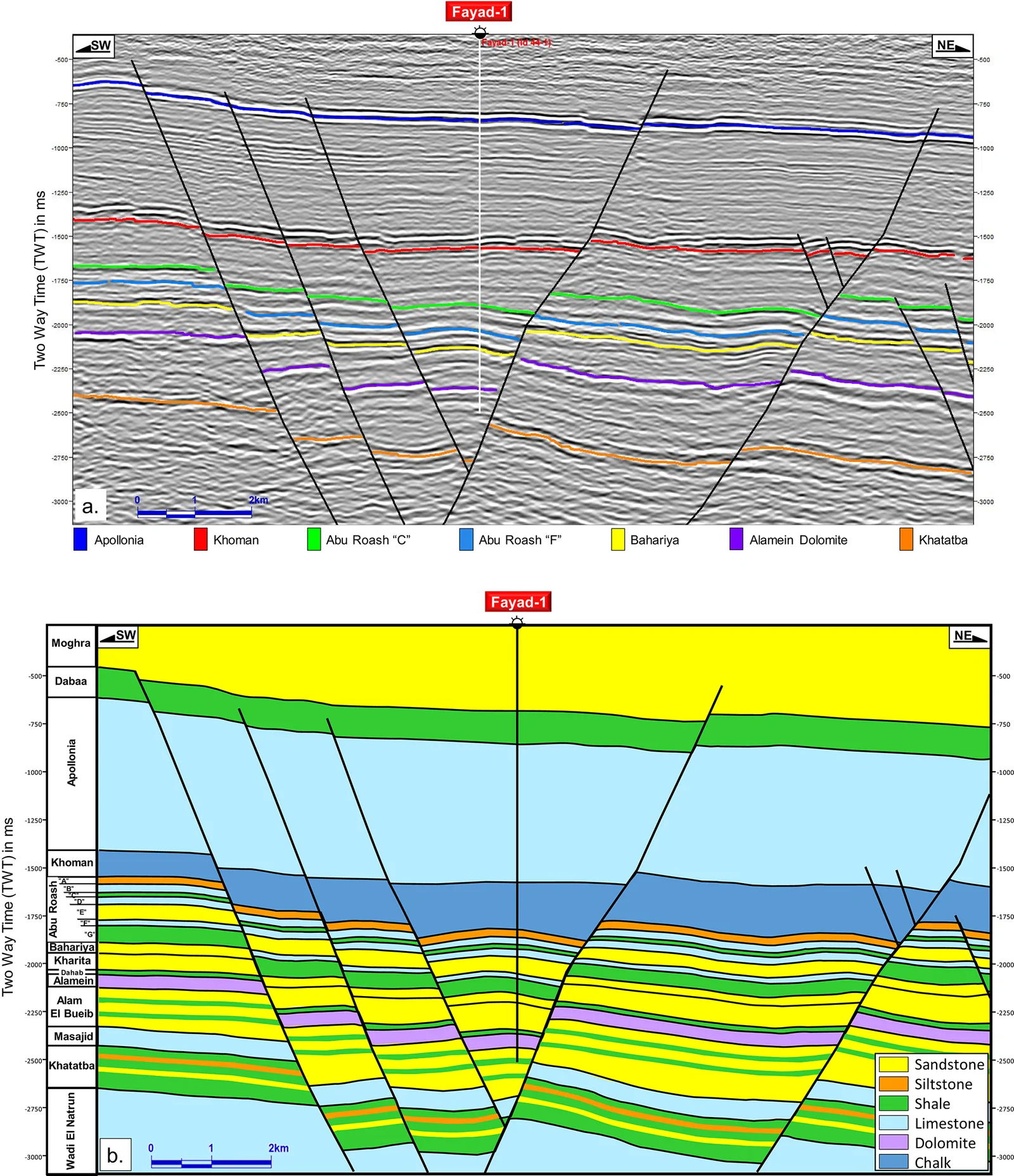
(**a**) Reinterpretation of NE–SW seismic profile passing through Fayad-1 well, (**b**) The NE–SW geo-seismic cross-section passing through Fayad-1 well, showing the dominant lithology of each horizon, located in Fig. 5.

**Fig. 10:**
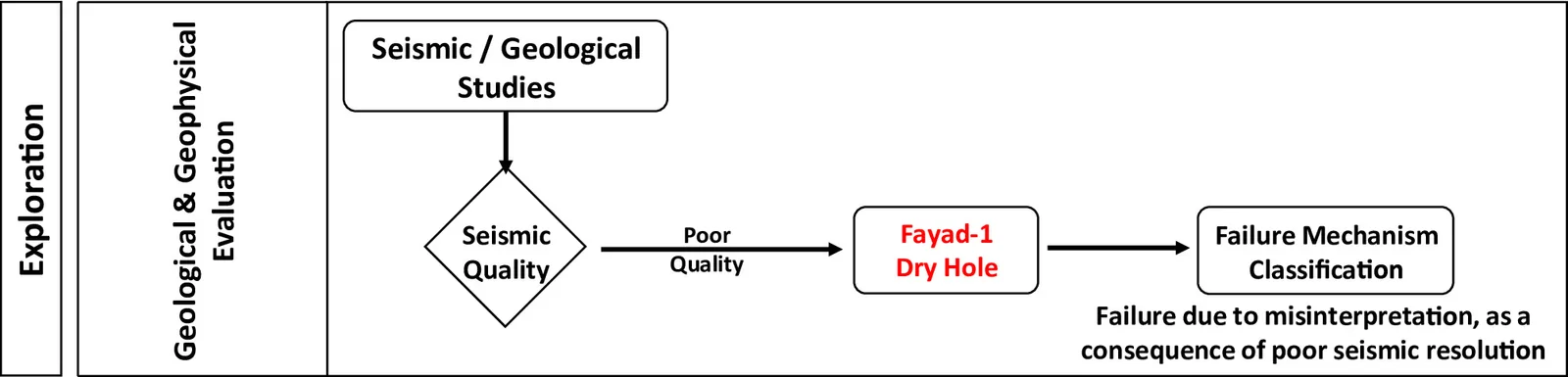
Poor seismic imaging led to drilling the Fayad-1 (Id 44 − 1) dry hole.

Subsequent re-evaluation, including a fluid-inclusion analysis and interpretation of newly available 3D seismic data, provides important insights into the cause of failure. Live oil was identified within Cretaceous intervals, confirming hydrocarbon migration and an active petroleum system. These results suggest that the lack of success in drilling well Fayad-1 cannot be attributed to charge failure, the explanation that had been suggested by^33^. Reinterpretation of the 3D seismic data reveals that the well was drilled out of the structure on the downthrown (hanging-wall) block rather than the targeted upthrown (footwall) closure (Fig. 9) because the well location was selected using legacy 2D seismic data with limited resolution, sparse spatial coverage, and poor fault imaging. Therefore, the Fayad-1 well is interpreted as structural (trap-definition) failure caused by incorrect well placement as a result of structural misinterpretation of low-quality legacy 2D seismic data (Fig. 10), Tables 5 and 6.

**Table 5:** Semi-quantitative geological risk assessment of Fayad-1 well, with the operational-performance risk assessment.

Risk element	Score	Description
Structure/trap integrity	1	Seismic reinterpretation showed the well was drilled on a downthrown (hanging-wall) block, missing structural closure
Reservoir quality	4	Good reservoir properties were present, with no evidence that reservoir quality was the main cause of failure
Seal effectiveness	4	Regional sealing intervals were present, with no evidence of seal failure
Source rock (charge and migration)	4	Fluid inclusion analysis proved hydrocarbon charge and migration
Operational performance	5	No major drilling problems found

The average geological risk is 3.25 (~ 3), reflecting a moderate geological risk.

**Table 6:** Semi-quantitative interpretation confidence assessment of Fayad-1 well.

Confidence element	Score	Description
Data quality	2	The original prospect was generated from legacy 2D seismic data, with low resolution
Data quantity and coverage	3	Moderate regional data coverage, as sparse 2D seismic coverage
Direct evidence	4	The interpretation is supported by direct subsurface evidence, including well data and fluid inclusion analysis

The average interpretation confidence is 3, reflecting moderate confidence in data.

The semi-quantitative assessment showed an average geological risk is 3.25 (~ 3) (moderate geological risk), with very low operational-performance risk (Table 5), while the average interpretation confidence is 3 (moderate confidence) (Table 6). Accordingly, the integrated exploration score for Fayad-1 is 3.25 × 3 = 9.75, corresponding to high exploration risk in the risk-confidence matrix (Fig. 8). The contrast between the generally favorable petroleum-system elements scores and the low structure/trap integrity identifies structural failure as the dominant cause of the dry hole. The moderate confidence score reflects the seismic interpretation uncertainty in data quality and coverage, due to reliance on legacy 2D seismic data during the original prospect evaluation. Overall, the integrated assessment supports the classification of Fayad-1 as a structural (trap-definition) failure.

### Dalia-1 well

Dalia-1 was drilled in 2009 to evaluate the hydrocarbon potential of a fault-bounded dip closure within a structurally complex zone of step-faulted highs between the Natrun and Alamein basins. The primary targets were the sandstone reservoirs of the Kharita, Alam El Bueib, and Khatatba formations; the secondary objective was any well-developed reservoir facies within the Abu Roash and Bahariya sections. Dalia-1 structure showed an elongated NW–SE-trending fault-bounded three-way dip closure with structural relief at the Bahariya, Alamein, and Wadi Natrun formations. The source rock was assumed to be the Middle Jurassic Khatatba Formation, which has been confirmed as the source rock in the East Tiba half-graben and its surroundings. The vertical sealing was expected to be provided by top Khatatba shales and intra-formational shale intervals within Alam El Bueib (AEB) and Kharita formations to seal the underlying reservoirs. The basal Bahariya carbonates were interpreted to seal the Kharita reservoir.

**Fig. 11:**
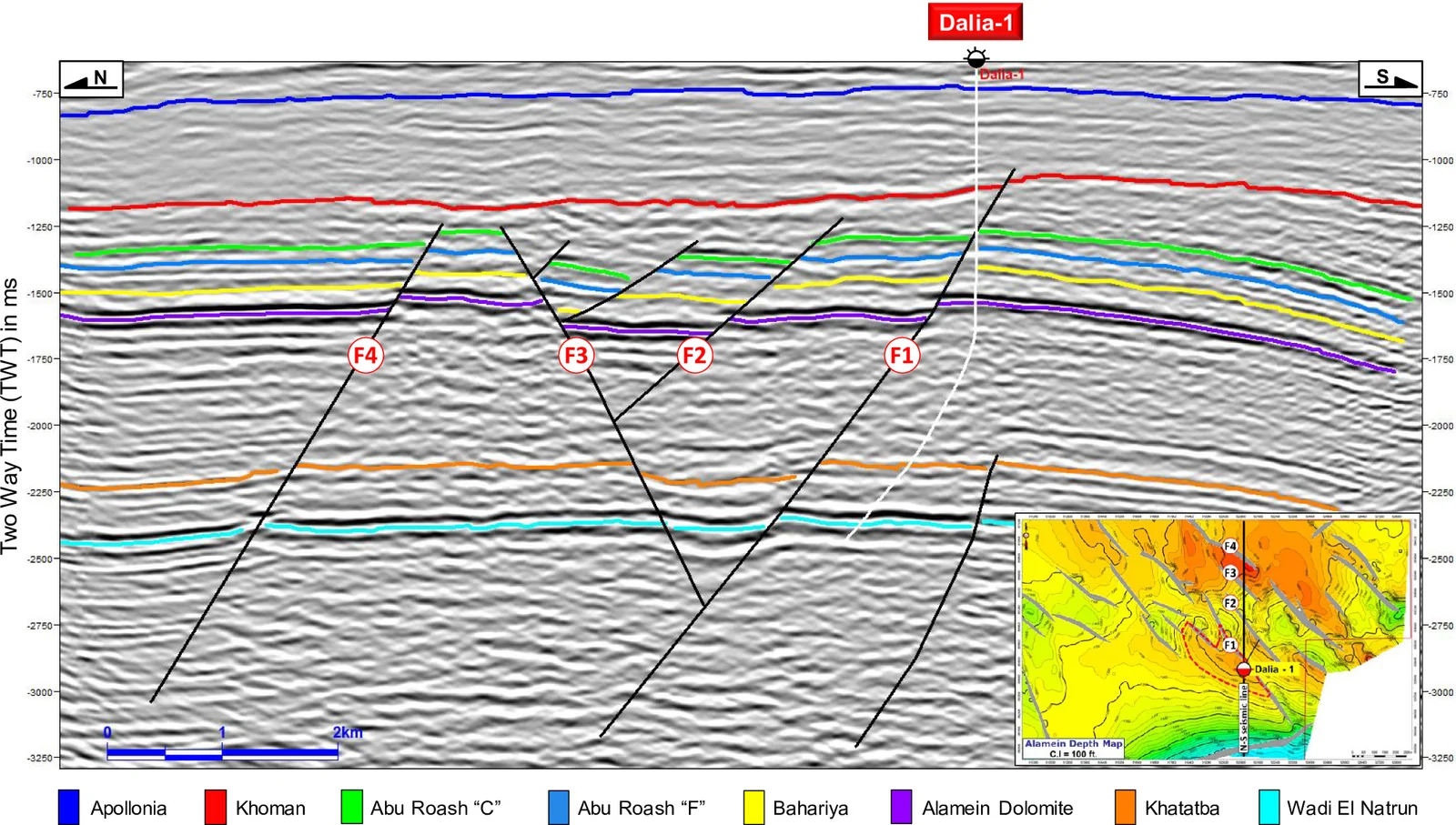
An interpretation of N–S TWT seismic profile passing through Dalia-1 well-1, located in Fig. 5.

The well reached a TD of 4,439 m (14,563 ft) in the Wadi El Natrun Formation (Fig. 11). Hydrocarbon shows (oil and gas) were encountered during drilling of the Alam El Bueib section, confirming the presence of an active petroleum system within the East Tiba half-graben. Petrophysical evaluation (Table 7), using the cut-off criteria summarized in Table 2, indicates that the primary clastic objectives, Bahariya, Kharita, and Alam El Bueib (AEB) reservoirs, were interpreted as water-bearing and no producible pay. Although the Kharita Formation has good reservoir properties with a net/gross ratio of 0.87 and an average effective porosity of 21%, it was completely water-saturated without net pay. This indicates a very good reservoir interval, but no hydrocarbon accumulation, suggesting the failure resulted from ineffective charge, migration, or incomplete sealing at the Kharita level rather than poor reservoir quality. Only a thin interval of ~ 4.5 m (14.8 ft) within the Alamein Formation showed possible pay, with 7% effective porosity, approximately 49% water saturation, low shale content (~ 2%), and positive oil shows; however, this interval was considered insufficient for a commercial discovery. Consequently, the well was plugged and abandoned.

**Table 7:** Summary of interpreted petrophysical properties for the Dalia-1well.

Reservoir	Gross thickness		Net reservoir		Net/Gross (*N*/G)	Net pay		Average effective porosity (PHIE) %	Average water saturation (Sw) %	Average shale volume (Vsh) %
	(m)	(ft)	(m)	(ft)		(m)	(ft)			
Kharita	182	598	159	522	0.87	0	0	21	100	15
Alamein	40	132	14	47	0.36	4.5	14.8	7	49	2
AEB	884	2900	521	1710	0.59	0	0	19	99	18
U. Safa	500	1641	4.6	15	0.01	0	0	15	71	13
L. Safa	54	178	6	20	0.16	0	0	17	80	14

**Fig. 12:**
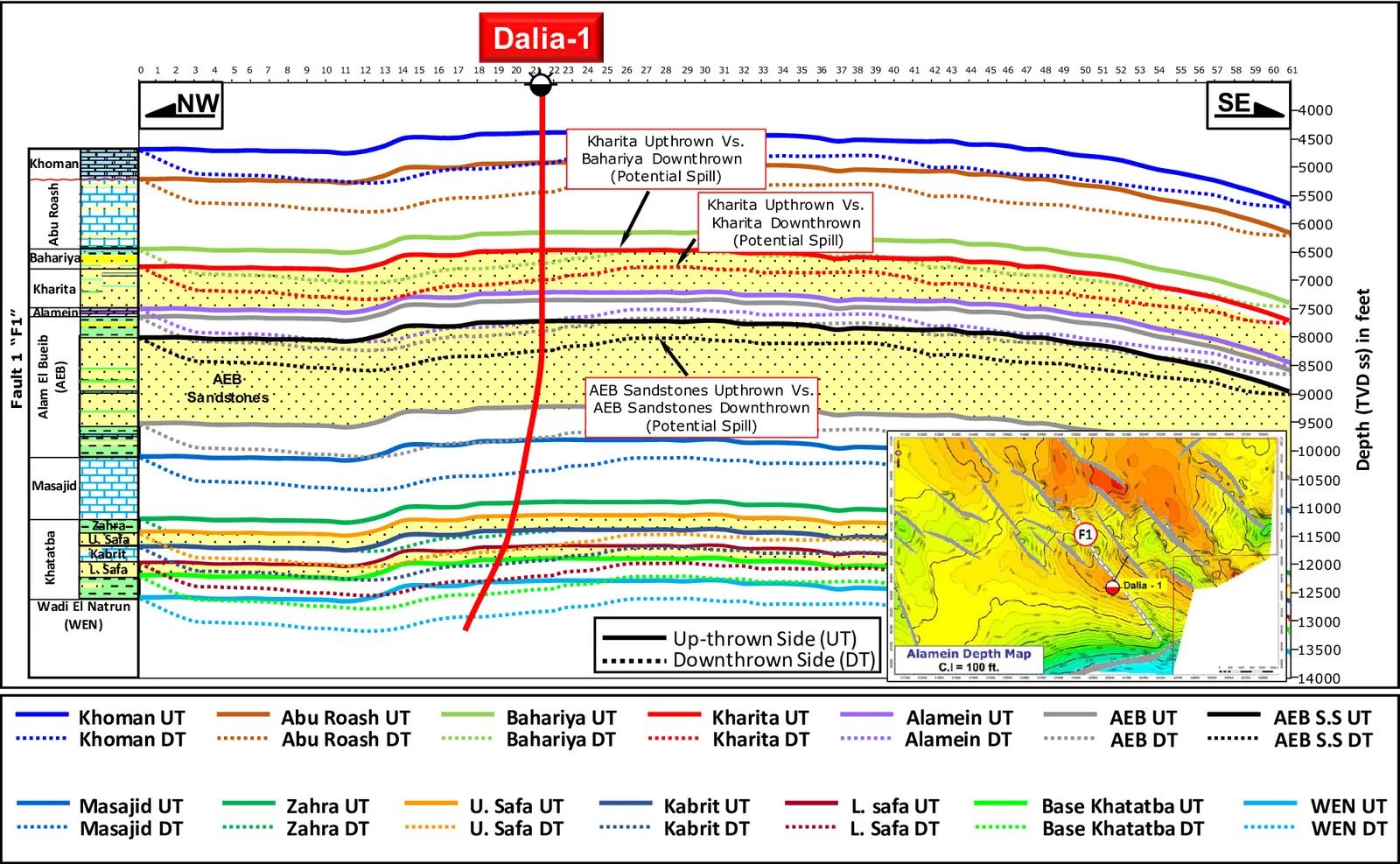
Fault seal analysis of the Dalia-1 prospect at the Alamein and Wadi El Natrun (WEN) maps, showing the NW–SE fault-bounded three-way dip closure and potential spill points due to the juxtaposition relationships across the major fault (F1); the upthrown block is represented by solid lines, while the downthrown block is represented by dashed lines.

The structural configuration of the Dalia-1 well indicates that trap definition was not the primary cause of failure. The well successfully penetrated the targeted fault-bounded closure, confirming the structural interpretation. However, fault seal analysis (Fig. 12) reveals several juxtaposed potential spill points along the major fault (F1) that may have reduced the trap efficiency through leakage or incomplete charge, affecting the Alamein and Wadi El Natrun formations.

**Fig. 13:**
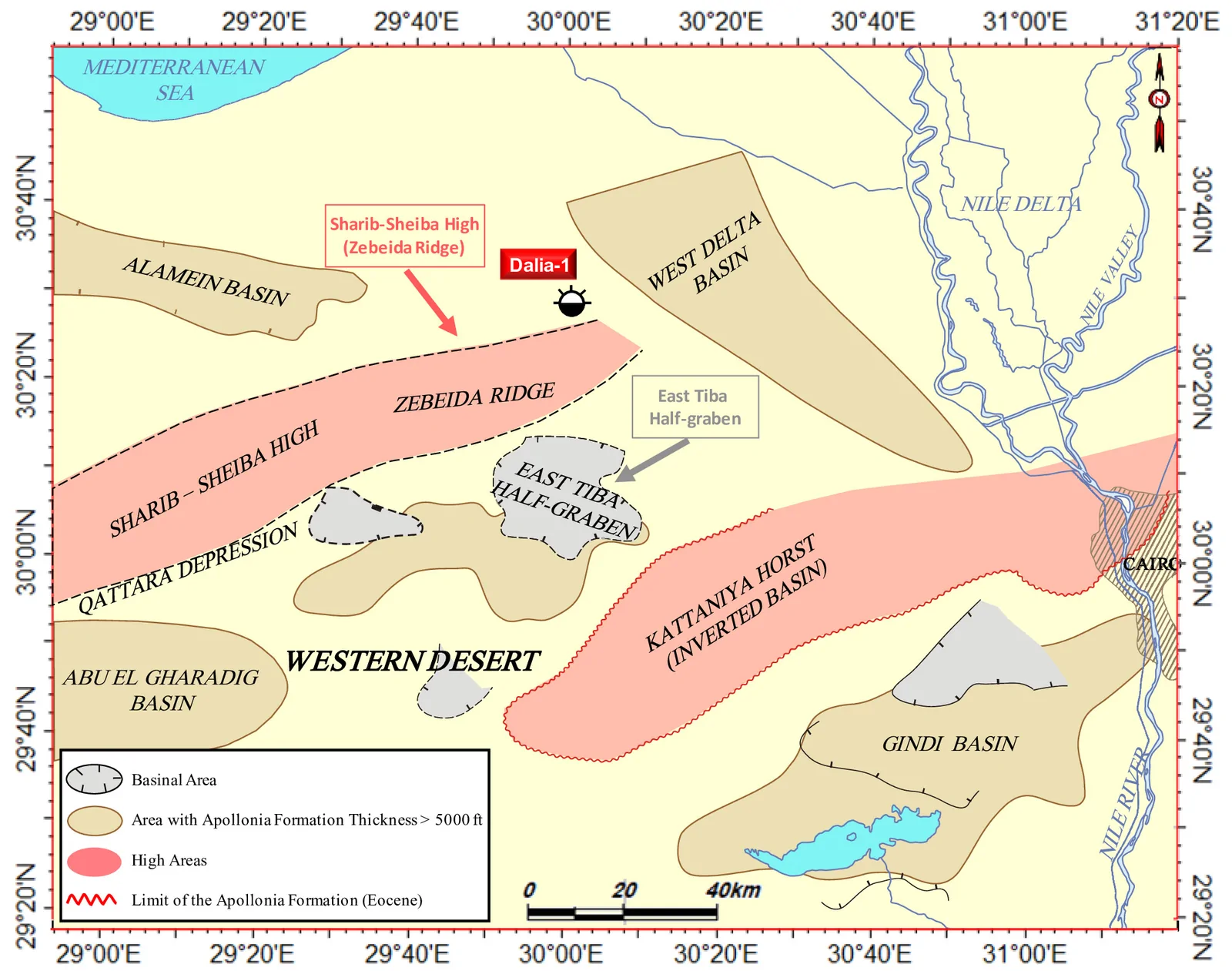
Schematic map highlights the Sharib-Sheiba High (locally the Zebeida Ridge) that prevents hydrocarbon migration from the East Tiba half‑graben to the Dalia-1 well.

**Fig. 14:**
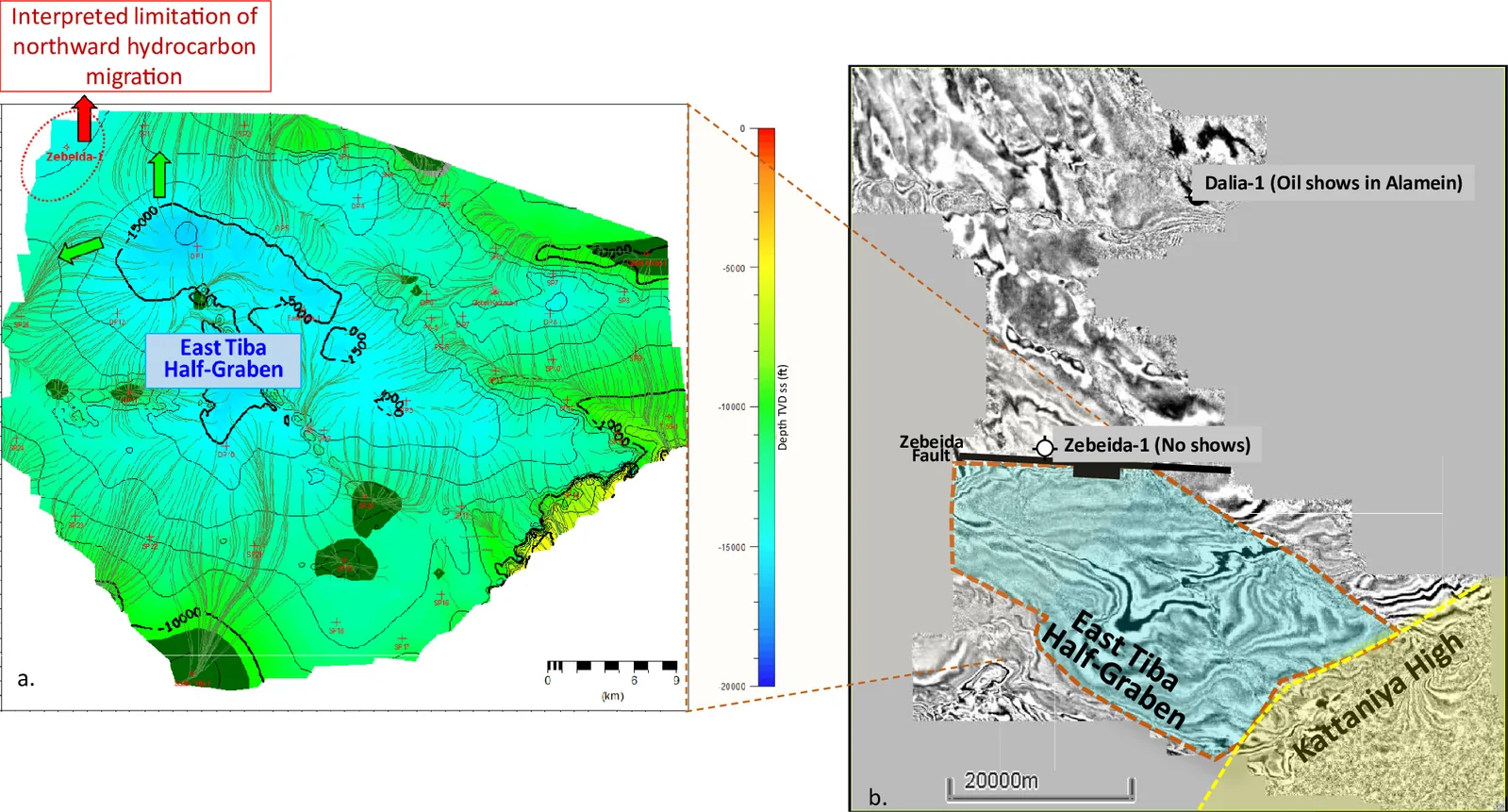
Regional structural framework supporting the interpreted migration-controlled failure of the Dalia-1 well. (**a**) Present-day Alamein depth contour map showing the structural configuration and interpreted migration pathways; (**b**) Time slice (TWT) at ~ 1580 ms (Alamein level), highlighting the structural margin of the East Tiba half-graben.

The Dalia-1 well is located north of the East Tiba half-graben, whereas the Zebeida Ridge, an extension of the Sharib-Sheiba High, forms a structural high along the northern margin of the interpreted Jurassic depocenter and hydrocarbon kitchen (Figs. 13 and 14), separating the southern East Tiba half-graben from the northern area by a series of old E-W-trending faults. This regional structural configuration, probably acting as a potential migration barrier, that may have restricted northward hydrocarbon movement from East Tiba half-graben toward the Dalia-1 structure, despite the presence of reservoir units. Figure 13 shows the regional schematic structural setting of the northeastern part of the Western Desert, illustrating the tectonic relationship between the East Tiba half-graben, Alamein Basin, Natrun Basin, and the Kattaniya High. The map highlights the Zebeida Ridge and the Sharib-Sheiba High, showing Dalia-1 structural position relative to the major regional tectonic elements controlling basin architecture in the northern Western Desert. Figure 14 provides additional support for the migration-controlled interpretation by showing the relationship between the Jurassic depocenter and the structural margin represented by the Zebeida Ridge.

**Fig. 15:**
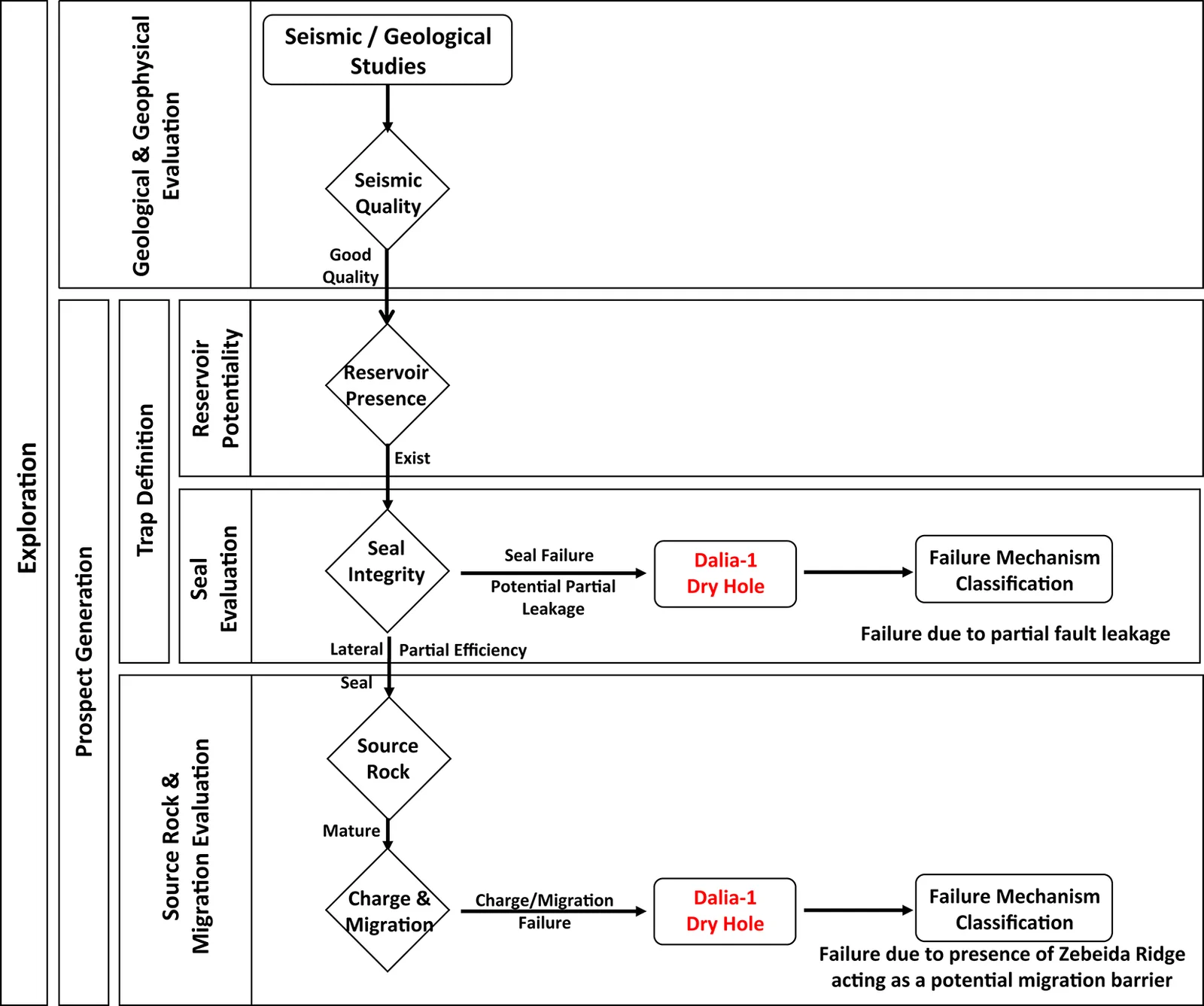
Migration-related failure led to drilling the Dalia-1 dry hole.

No hydrocarbon shows were recorded in the Zebeida-1 well, while only weak oil shows were encountered in the Alamein Formation at the Dalia-1 well, possibly indicating limited charge from alternative migration pathways from Natrun or Alamein basins. This observation, with the fault-seal juxtaposition analysis (Fig. 12), suggests that the hydrocarbon charge reaching the Dalia-1 closure was insufficient to establish a commercial accumulation. Although no regional basin modeling was performed, the integrated structural, drilling, petrophysical, and fault-seal evidence supports a migration-controlled failure, with possible fault-seal inefficiency acting as a secondary contributing factor (Fig. 15).

The structural configuration suggests that, despite successful trap definition and drilling of the closure, regional migration barriers may have limited hydrocarbon charge and reduced hydrocarbon accumulation within the prospect, affecting the failure of Dalia-1.

**Table 8:** Semi-quantitative geological risk assessment of Dalia-1 well, with the operational-performance risk assessment.

Risk element	Score	Description
Structure/trap integrity	4	Well-defined three-way fault-bounded closure was successfully penetrated without evidence for trap failure
Reservoir quality	4	Good reservoir properties were present, especially Kharita sandstones but without net pay due to high water saturation
Seal effectiveness	3	Regional top sealing intervals were present and appear effective, but fault-seal analysis shows potential spill points and possible lateral leakage along the major fault
Source rock (charge and migration)	3	Mature Jurassic Khatatba source rock within the East Tiba half-graben and surrounding area; oil and gas shows encountered while drilling the Alam El Bueib interval confirm active petroleum system, but the regional migration pathways appear restricted by regional structural configuration, reducing the migration efficiency
Operational performance	5	The well was successfully drilled and logged without major operational problems

The average geological risk is 3.5 (~ 4), reflecting a low geological risk.

**Table 9:** Semi-quantitative interpretation confidence assessment of Dalia-1 well.

Confidence element	Score	Description
Data quality	4	3D seismic data integrated with well logs and petrophysical evaluation provide good definition for structure and reservoir
Data quantity and coverage	3	3D seismic coverage and regional offset wells provide relative data coverage for structural and geological interpretation
Direct evidence	4	The interpretation is supported by direct well data, petrophysical evaluation, drilling shows, and fault-seal analysis

The average interpretation confidence is 3.67 (~ 4), reflecting a high confidence in data.

The semi-quantitative assessment showed an average geological risk of 3.5 (~ 4), reflecting low geological risk (Table 8), while the average interpretation confidence is 3.6 (~ 4), reflecting high confidence (Table 9). Accordingly, the integrated exploration score for Dalia-1 is 3.5 × 3.67 = 12.8, corresponding to moderate exploration risk in the risk-confidence matrix (Fig. 8). The generally favorable scores of trap integrity, reservoir quality, and operational performance indicate that these elements were not the main cause of failure. The low scores assigned to source rock (charge and migration) efficiency and seal effectiveness reveal that restricted hydrocarbon charge and possible fault-seal inefficiency are the main geological uncertainties. This interpretation is supported by the fault seal analysis, which indicates ineffective lateral fault sealing and potential leakage pathways, consistent with integrated seismic and fault seal studies from the northern part of the Western Desert demonstrating the critical role of fault sealing in hydrocarbon preservation^36^. Although oil and gas shows confirm an active petroleum system, the structural high acting as a potential barrier related to the Zebeida Ridge restricted hydrocarbon migration toward the prospect, which may have contributed to the dry-hole result. Therefore, the integrated assessment supports the classification of Dalia-1 as a migration-controlled failure, with possible partial fault-seal failure as a secondary contributing factor despite favorable reservoir properties in the Kharita Formation.

### Thanaa-1X well

Thanaa-1X was designed to test the hydrocarbon potential in the southeastern part of the study area, east of the Qattara Depression, within a structurally intermediate zone bounded to the north by the Alamein Basin, to the west by the Abu Gharadig Basin, and to the southeast by a flank near the margin of the East Tiba half-graben. The targets were Jurassic reservoirs, with the up-dip migration pathway from the kitchen through the same reservoir expected to charge them. The primary objective was the sandstone reservoir of the Khatatba Formation (Upper and Lower Safa Members), while the secondary target was the sandstone of the Bahrein Formation.

**Fig. 16:**
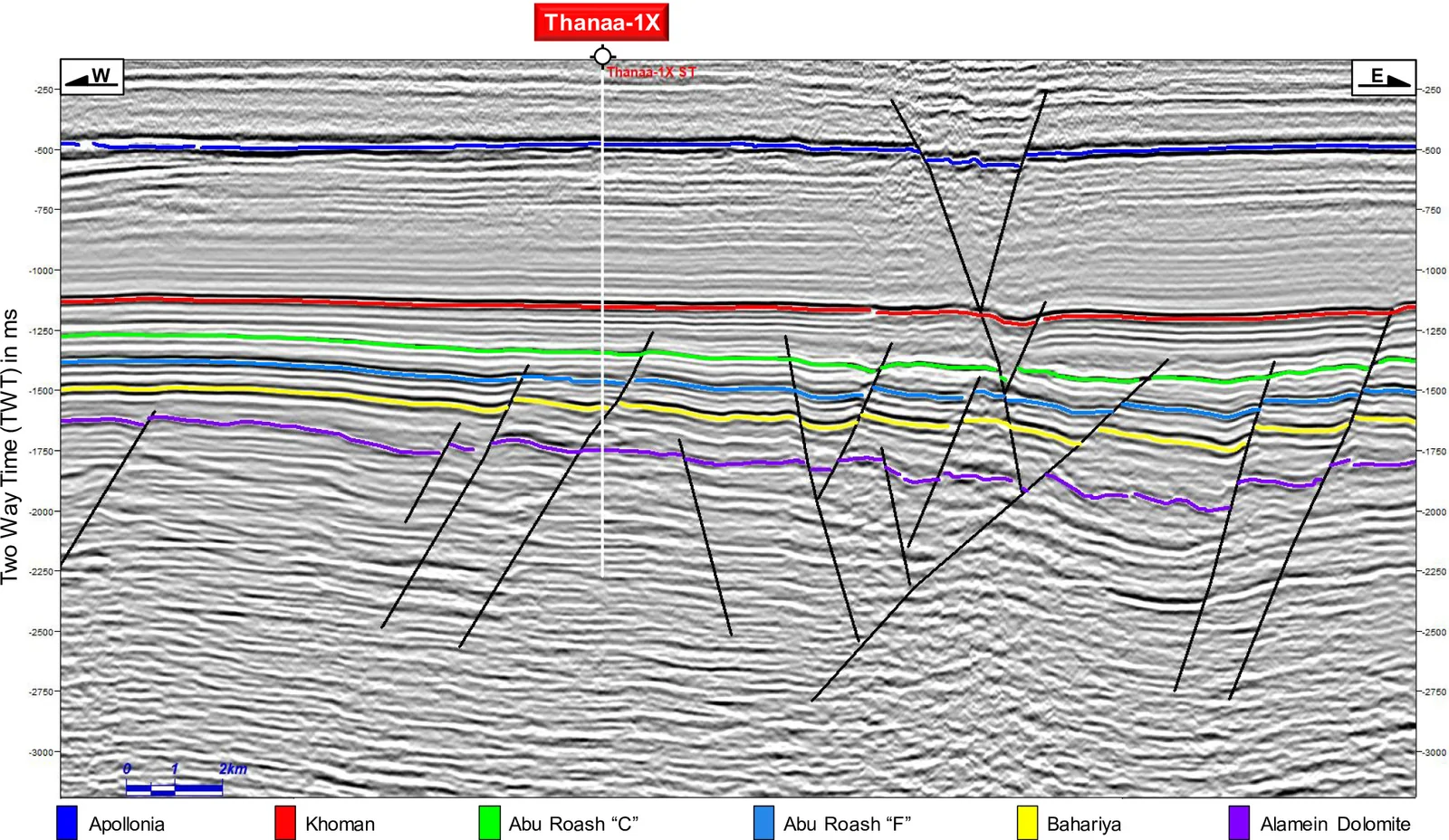
An interpretation of E-W TWT seismic profile passing through Thanaa-1X well, located in Fig. 5.

Thanaa-1X was drilled in 2010, and during drilling, the drill string became stuck between 3002 and 3163 m measured depth (MD) (9849 to 10,377 ft MD) within the Kharita Formation, prompting placement of a cement plug and sidetracking of the well to Thanaa-1X ST. The sidetrack reached a TD of 4,425 m MD (-4254 m TVDss) ~ (14,518 ft MD (–13,957 ft TVDss)) within the Bahrein Formation (Fig. 16). Weak oil shows were reported through several stratigraphic intervals, including the Cretaceous Abu Roash “E”, “G”, Upper Bahariya, dolomite of Alamein Formation, and Jurassic Upper and Lower Safa sandstones.

The petrophysical evaluation (Table 10) using the cut-off criteria summarized in Table 2 has been carried out for the Upper and Lower Bahariya, Kharita, Dahab, Alamein, AEB, Masajid, Zahra, U. Safa, Kabrit, L. Safa, and Bahrein formations. Petrophysical interpretation indicated limited scattered net pay, approximately 0.5 m (1.6 ft) in the Lower Safa sandstone reservoir and up to 1.5 m (5 ft) in the Alamein dolomite reservoir, with an average porosity ranging between 8 and 15%. However, these pay intervals were detected from the petrophysical evaluation of the 6” hole and were not confirmed by oil or gas shows in the mud log. These scattered net pay intervals were characterized by high water saturation, which was insufficient for a commercial well announcement. Due to the poor quality of the 6” wireline data, which was related to poor hole conditions and the lack of sufficient LWD logs, these pay intervals are considered not real. In contrast, no pay was detected in the Lower Bahariya, Kharita, Alam El Bueib (AEB), and Bahrein reservoirs. Accordingly, the well was classified as a dry hole.

**Table 10:** Summary of interpreted petrophysical properties for the Thanaa-1X.

Reservoir	Gross thickness		Net reservoir		Net/Gross (*N*/G)	Net pay		Average effective porosity (PHIE) %	Average water saturation (Sw) %	Average shale volume (Vsh) %	Shows
	(m)	(ft)	(m)	(ft)		(m)	(ft)				
A/R “E”	65	213	–	–		0.45	1.5	23	48	–	V. poor
U. Bahariya	87	285	15	49	0.17	1.05	3.44	13	99	6	V. poor
Kharita	159	522	66	217	0.42	0	0	14	99	8	
Alamein	56	184	4	13	0.07	1.5	4.9	12	36	2	V. poor
AEB	358	1175	222	728	0.62	0	0	13	100	7	
U. Safa	292	958	8	26	0.03	1.05	3.4	14	39	17	
L. Safa	199	653	0.5	1.6	0.002	0.5	1.64	9	22	15	
Bahrein	67	220	1.9	6	0.03	0	0	9	95	0	

**Fig. 17:**
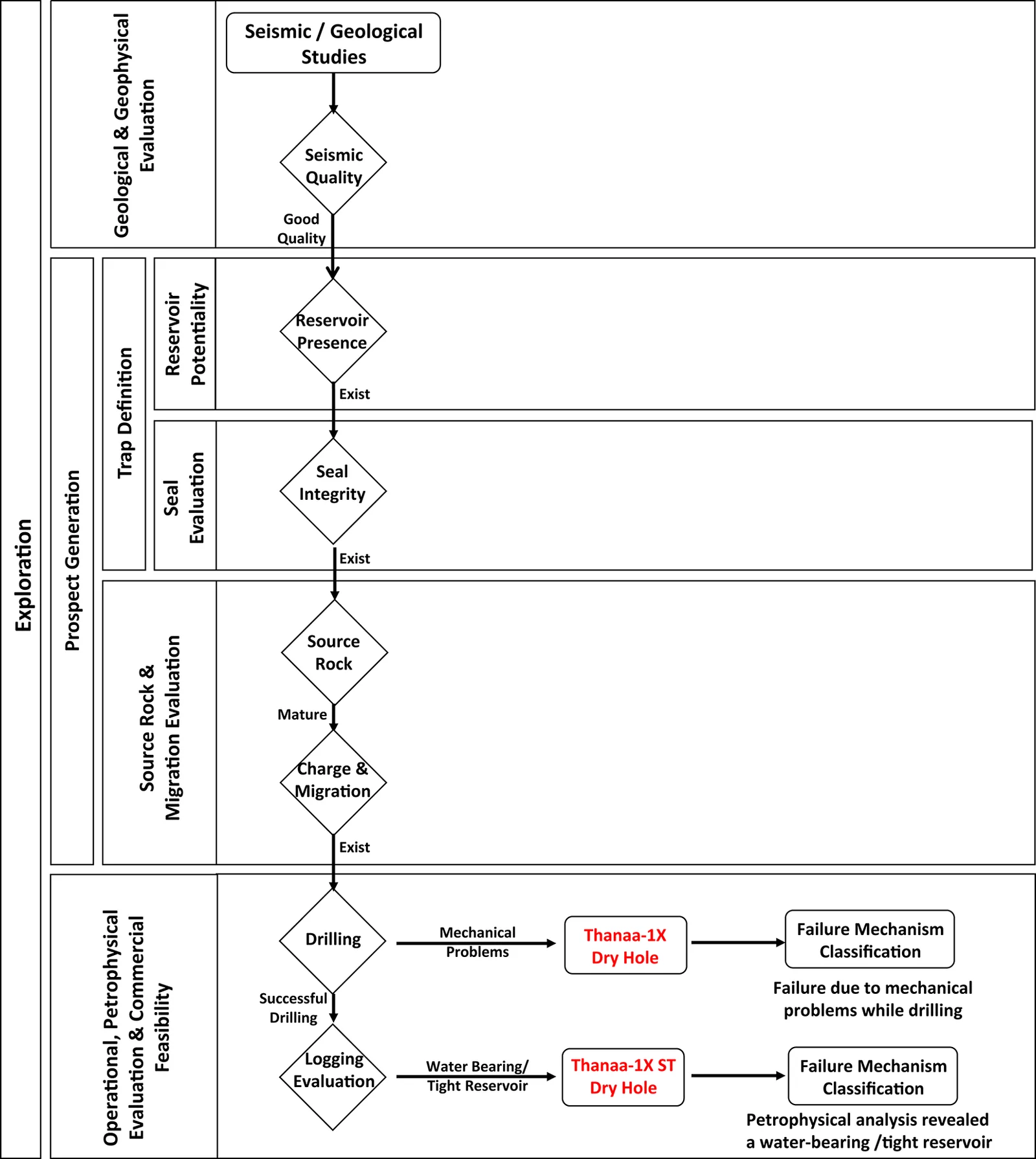
Schematic illustration of the different failure mechanisms at Thanaa-1X (operational-related failure in the original borehole) and reservoir-related failure in the Thanaa-1X ST dry hole.

The occurrence of weak oil shows across multiple Cretaceous and Jurassic stratigraphic levels indicates an effective petroleum system. However, the absence of commercially significant pay indicates that hydrocarbons did not accumulate in sufficient quantities. The two well trajectories therefore represent different failure mechanisms. The original Thanaa-1X borehole faced operational failure due to stuck drill string without reaching the main objectives, while the Thanaa-1X ST successfully reached the main targets but with inadequate reservoir properties. Accordingly, the Thanaa-1X well is interpreted as a dry hole, where hydrocarbons reached the structure but did not accumulate in commercial quantities. This example emphasizes the challenges of predicting facies changes and diagenetic processes in the different reservoirs within a structurally segmented half-graben system. The main cause of failure is interpreted as an operational failure for the original Thanaa-1X well, while a reservoir-related failure is interpreted in the sidetrack (ST) option of the well (Fig. 17).

**Table 11:** Semi-quantitative geological risk assessment of Thanaa-1X ST well, with operational-performance risk assessment.

Risk element	Score	Description
Structure/trap integrity	4	The sidetrack successfully penetrated the structural closure with no evidence of structural mispositioning
Reservoir quality	2	The reservoir thicknesses were thin, discontinuous, and the net pay intervals show high water saturation, low to moderate effective porosity
Seal effectiveness	4	Regional sealing intervals are present, with no direct evidence of seal failure
Source rock (charge and migration)	4	Weak oil shows were recorded in Cretaceous to Jurassic reservoirs, indicating hydrocarbon migration into the area, with no commercial accumulation
Operational performance	5	The sidetrack was successfully drilled and logged without major operational problems

The average geological risk is 3.5 (~ 4), reflecting a low geological risk in the Thanaa-1X ST well. Moreover, drilling of the original Thanaa-1X well was interrupted by a stuck drill string, preventing the well from reaching the targeted Jurassic reservoirs. Consequently, the original hole was interpreted as having a very high operational risk, indicating the failure mechanism in the original Thanaa-1X is drilling performance, rather than geological conditions.

**Table 12:** Semi-quantitative interpretation confidence assessment of Thanaa-1X ST well.

Confidence element	Score	Description
Data quality	2	3D seismic data integrated, but the 6” wireline logs were affected by the pore-hole conditions, with insufficient LWD data reducing confidence in the petrophysical evaluation
Data quantity and coverage	3	3D seismic coverage, mud logs, and petrophysical data were available, with limited logs data coverage for detailed reservoir evaluation
Direct evidence	3	Direct well and mud logs data, with weak oil shows, but potential pay intervals were not confirmed by other data to support the petrophysical evaluation

The average interpretation confidence is 2.67 (~ 3), reflecting moderate confidence in the data.

The semi-quantitative assessment showed an average geological risk of 3.5 (~ 4), reflecting low geological risk (Table 11), while the average interpretation confidence of 2.67 (~ 3), reflects moderate confidence (Table 12). Accordingly, the integrated exploration score for Thanaa-1X ST is 3.5 × 2.67 = 9.3, corresponding to high exploration risk in the risk-confidence matrix (Fig. 8). The generally favorable scores of structure/trap integrity, source rock (charge and migration), and seal effectiveness indicate that these elements were not the main cause of the dry hole, whereas reservoir quality received the lowest geological score in the Thanaa-1X ST indicating the main geological uncertainty. On the other hand, the operational performance received the lowest score in the original hole of Thanaa-1X, which contributed to the dry-hole result. Therefore, the integrated assessment supports the classification of Thanaa-1X ST as a reservoir-related failure. In contrast, the original Thanaa-1X borehole represents an operational failure because the stuck drilling string.

## Discussion

The analysis of three dry exploratory wells in the East Tiba-Natrun study area reveals that the exploration failure resulted from different geological (reservoir- and migration-related), geophysical (structural interpretation), and operational (drilling/mechanical) failure mechanisms, rather than from a single petroleum-system failure, despite their location within a proven hydrocarbon province. The integrated assessment identifies structural definition as the dominant failure mechanism at Fayad-1, restricted charge delivery with possible fault-seal leakage at Dalia-1, operational failure while drilling Thanaa-1X original borehole, and reservoir-related failure in the Thanaa-1X ST well. These contrasting results demonstrate that the studied dry holes cannot be attributed to the same regional inactive petroleum system, and are consistent with exploration challenges previously documented across the northern Western Desert, where structural uncertainty, reservoir heterogeneity, and migration efficiency commonly influence exploration success.

The Fayad-1 well shows the effect of seismic imaging quality on structural definition and well placement. Reinterpretation using new available 3D seismic data showed that the well was drilled outside the intended closure on the downthrown (hanging-wall) block, whereas the original location had been selected using legacy 2D seismic data with poor resolution and low coverage. This case highlights the critical importance of acquiring high-resolution 3D seismic data to enhance subsurface structural imaging and optimize trap definition in the complex structural settings of the Western Desert. The results demonstrate that dry-hole reinterpretation using new seismic data can significantly improve geological understanding and reduce structural uncertainty during future prospect generation.

Geologic-related failures encompass poor reservoir quality and reservoir heterogeneity. Available petrophysical data analysis (Table 13) indicates variable reservoir quality, with effective porosity ranging from 7 to 21% and considerable differences in net reservoir thickness among the evaluated intervals. Although hydrocarbon shows were recorded in the Abu Roash “E” and Alamein reservoirs, no commercial flow was obtained. This suggests that reservoir performance may have been influenced by reservoir heterogeneity, hydrocarbon distribution, or trapping efficiency rather than reservoir presence alone. The observed variability in reservoir quality is consistent with previous studies, which show significant vertical and lateral heterogeneity within Abu Roash reservoirs in the Western Desert of Egypt, highlighting the importance of integrated petrophysical and geological evaluation for reservoir characterization^39^. However, core analysis and permeability measurements were unavailable, preventing direct quantitative evaluation of permeability effects. Therefore, reservoir heterogeneity and variability in the petrophysical properties are interpreted as significant contributing factors to the non-commercial well. Nevertheless, the reservoir quality varied among wells and intervals, demonstrating that reservoir characteristics alone cannot explain exploration failure. The integrated geological, petrophysical, and petroleum–system analyses indicate that the dominant dry-hole mechanism changed from one well to another within the studied wells. The trap integrity represents one of the main geological risks in structurally controlled plays of the Western Desert, where fault sealing is the main risk mechanism on hydrocarbon preservation^40^.

**Table 13:** Petrophysical summary data of all analyzed wells.

Well name	Reservoir	Gross thickness		Net reservoir		Average effective porosity (PHIE) %	Hydrocarbon shows
		m	ft	m	ft		
Fayad-1	Bahariya	124	407	N/A		3*	N/A
	Kharita	320	1050	N/A		9*	N/A
	Alamein	70	229	N/A		1.5*	N/A
	AEB	394	1293	N/A		6*	N/A
Dalia-1	Kharita	182	598	159	522	21	Absent
	Alamein	40	132	14	47	7	Absent
	AEB	884	2900	521	1710	19	V. weak oil shows
	U. Safa	500	1641	4.6	15	15	Absent
	L. Safa	54	178	6	20	17	Absent
Thanaa-1X ST	Alamein	56	184	4	13	12	V. weak oil shows
	U. Safa	292	958	8	26	14	Absent
	L. Safa	199	653	0.5	1.6	9	Absent

*Old data (after^40^).

Seal-integrity failure, such as a breached cap rock or a problem with sealing juxtaposition, can lead to hydrocarbon leakage. Migration-related failure represents another important risk identified in this study. The Dalia-1 well indicates that the presence of mature source rocks and hydrocarbon shows alone does not ensure commercial accumulations. The integrated structural interpretation, migration-pathways analysis, and fault-seal assessment suggest that hydrocarbon charge toward the Dalia structure was restricted by the regional tectonic configuration. The Jurassic rifting created the principal Khatatba source kitchen areas, such as within the East Tiba half-graben, whereas subsequent Late Cretaceous inversion generated structural highs and potential migration barriers that influenced hydrocarbon migration and trap filling. Consequently, Dalia-1 is interpreted as a migration-related failure, highlighting the importance of data collection, regional structural evolution, migration-pathways analysis, and fault-seal evaluation during prospect assessment. These observations highlight the relationship between basin evolution and petroleum-system elements and processes in the Western Desert.

The Thanaa-1X ST well represents a reservoir-related failure, as it has a water-bearing reservoir despite evidence of hydrocarbon shows. This case highlights the importance of building a robust geological facies distribution and porosity model to obtain results matching the expectations. Similarly, the operational failure encountered in the original Thanaa-1X borehole demonstrates that drilling-related problems can prevent adequate evaluation of prospective geological targets. Therefore, exploration success depends not only on geological understanding but also on effective drilling execution and well placement.

Operational drilling or mechanical failures consist of drilling issues (e.g., stuck drilling pipes, increased losses, loss of circulation, and hole instability), missing the target zone due to errors in directional drilling accuracy, and finally, logging/testing issues, such as errors in identifying the productive reservoir intervals. According to the poor quality of the wireline data due to poor hole conditions and the lack of sufficient logging while drilling (LWD) data, the interpreted pay intervals should be considered with caution. These operational uncertainties should be integrated with exploration-risk assessment and geological and geophysical uncertainties.

Overall, the results demonstrate that exploration failure should not be interpreted simply as petroleum–system failure. Instead, unsuccessful wells may result from different combinations of geological, geophysical, and operational uncertainties. Consequently, identifying the dominant failure mechanism for each dry hole provides valuable information for refining geological models and reducing the probability of repeating similar exploration failures in future drilling.

The scientific contribution of this study demonstrates the value of applying a unified dry-hole assessment workflow to multiple wells. Each well was evaluated using the same geological, geophysical, petrophysical, drilling, and petroleum-system criteria to identify and rank the dominant exploration risks according to their relative influence on the exploration success (Table 14). The comparative analysis indicates that exploration failure results from a combination of structural uncertainty, trap integrity issues, reservoir quality limitations, and charge-related risks across the studied wells. The cross-well comparison reveals the relative importance of these factors and provides insights that are not obvious from individual single-well evaluations. These results emphasize the importance of evaluating all petroleum-system elements collectively during prospect maturation rather than relying on individual risk factors.

In addition, the results highlight the impact of seismic imaging quality on prospect definition and exploration success. Wells evaluated using legacy 2D seismic data were subject to structural uncertainty, showing how limitations in seismic subsurface imaging can contribute to unsuccessful drilling results. These observations support the use of new 3D seismic data, integrated interpretation workflows, migration-pathways analysis, and fault-seal assessment to improve prospect evaluation and reduce structural risk.

The significance of this work lies in the cross-well comparison of three dry holes within a common geological framework (Table 15), the identification and ranking of common exploration risks (structural, reservoir, seal, and charge-related factors), and the development of a practical decision-tree workflow for prospect selection. These insights provide a transferable methodology for improving prospect evaluation and reducing exploration uncertainty in the Western Desert and may also apply to other petroleum provinces worldwide, where exploration success depends on the interaction between structural evolution, petroleum-system elements, and data quality.

**Table 14:** Descriptive and semi-quantitative cross-well comparison for geological risk/interpretation confidence elements.

Geological risk	Fayad-1		Dalia-1		Thanaa-1X ST	
	S	*R*	S	*R*	S	*R*
Trap integrity	**1**	V. High	4	Low	4	Low
Reservoir quality	4	Low	4	Low	**2**	High
Seal effectiveness	4	Low	**3**	Moderate	4	Low
Source rock	4	Low	**3**	Moderate	4	Low
Average geological risk score ≈	3.25		3.5		3.5	

Interpretation confidence	S	C	S	C	S	C
Data quality	**2**	Low	4	High	**2**	Low
Quantity and coverage	3	Moderate	3	High	3	Moderate
Direct evidence	4	High	4	High	3	Moderate
Average interpretation confidence score ≈	3		3.67		2.67	
**Integrated exploration score**	9.75		12.85		9.3	
	High		Moderate		High	

Where S is Score, R is Risk, and C is Confidence.

Table 15 briefly shows the overall dominant cause of failure for the three analyzed wells. Cross-well analysis is valuable because it demonstrates that not all dry holes failed for the same reason, allowing identification and ranking of exploration risks across the study area.

**Table 15:** Cross-well comparison for the dominant failure type with the average scoring.

Well	Geological risk score	Confidence score	Integrated exploration score	Drilling performance score	Risk category	Dominant failure type
Fayad-1	3.25	3	9.75	5	High	Structural definition/seismic resolution failure
Dalia-1	3.5	3.67	12.85	5	Moderate	Migration/charge barriers and sealing capacity
Thanaa-1X	–	–	–	1	–	Operational/drilling failure due to stuck pipes
Thanaa-1X ST	3.5	2.67	9.3	5	High	Reservoir quality/limited net pay thickness

Fayad-1 failed due to trap-definition uncertainty, whereas Dalia-1 and Thanaa-1X encountered active petroleum systems but failed: Dalia-1 due to charge-related failure and partially sealing integrity, whereas Thanaa-1X original hole failed because of an operational problem, while the sidetrack Thanaa-1X ST mostly because of inadequate reservoir performance and non-commercial hydrocarbon accumulations.

## Conclusion

This study evaluated the causes of failure in three exploratory wells drilled in the Western Desert of Egypt, using an integrated dry-hole analysis workflow based on petroleum-system evaluation. The results reveal that the integrated seismic interpretation, well data, geochemical analysis, petrophysical evaluation, and operational drilling data provide a systematic approach for identifying the geological, geophysical, and operational factors contributing to the exploration failure.

The analysis shows that successful exploration requires integrated evaluation of seismic imaging quality, petroleum-system elements, and structural evolution rather than relying mainly on structural closure. The analyzed dry holes reveal that exploration failure in the East Tiba-Natrun area is controlled mainly by structural uncertainty, migration-related risks, reservoir quality, and operational limitations, rather than source-rock effectiveness. In a structurally complex setting, the migration-pathway analysis, basin modeling, and fault-seal assessment should be integrated and evaluated before drilling to validate hydrocarbon charge and accumulation.

The results demonstrate the value of reinterpreting legacy dry wells using new seismic data, which can provide valuable geological understanding that can reduce exploration uncertainty and improve future decisions. Furthermore, integrated sedimentological and petrophysical analysis, supported by well correlations and seismic attributes, is essential for predicting reservoir distribution and quality to mitigate the reservoir-related uncertainty. While careful drilling planning and operational risk management remain necessary to minimize mechanical problems that may result in missing the target structure, or prevent successful testing of exploration targets and hence drilling a dry hole.

This study extends beyond the evaluation of individual dry holes by establishing a comparative framework for exploration failure analysis in the Western Desert of Egypt. The results identify the dominant causes of failure, rank the most significant exploration risks through a semi-quantitative assessment, and provide a practical decision-tree workflow that can be incorporated into future prospect selection and ranking. Therefore, the workflow presented in this study is not only helpful for post-drilling evaluation but also a practical guide for pre-drilling risk assessment, supporting well placement strategies to reduce uncertainty and improve exploration success in the Western Desert and comparable hydrocarbon basins.

Finally, exploration success can be significantly improved by shifting from isolated data interpretation to a fully integrated evaluation through the workflow approach. This confirms that all critical risk assessments convert dry-hole analysis into a powerful tool for future prospectivity.

## Data Availability

The data supporting the results of this study were obtained from the Egyptian General Petroleum Corporation. Data are, however, available from the corresponding author upon reasonable request and with permission of the Egyptian General Petroleum Corporation.
